# RanDeL-Seq: a High-Throughput Method to Map Viral *cis*- and *trans*-Acting Elements

**DOI:** 10.1128/mBio.01724-20

**Published:** 2021-01-19

**Authors:** Timothy Notton, Joshua J. Glazier, Victoria R. Saykally, Cassandra E. Thompson, Leor S. Weinberger

**Affiliations:** a Gladstone Center for Cell Circuitry, Gladstone Institutes, San Francisco, California, USA; b University of California, Berkeley, San Francisco Joint Graduate Group in Bioengineering, San Francisco, California, USA; c Department of Biochemistry and Biophysics, University of California San Francisco, San Francisco, California, USA; Duke University Medical Center

**Keywords:** biotechnology, human immunodeficiency virus, synthetic biology

## Abstract

It has long been known that noncoding genomic regions can be obligate *cis* elements acted upon in *trans* by gene products. In viruses, *cis* elements regulate gene expression, encapsidation, and other maturation processes, but mapping these elements relies on targeted iterative deletion or laborious prospecting for rare spontaneously occurring mutants. Here, we introduce a method to comprehensively map viral *cis* and *trans* elements at single-nucleotide resolution by high-throughput random deletion. Variable-size deletions are randomly generated by transposon integration, excision, and exonuclease chewback and then barcoded for tracking via sequencing (i.e., random deletion library sequencing [RanDeL-seq]). Using RanDeL-seq, we generated and screened >23,000 HIV-1 variants to generate a single-base resolution map of HIV-1’s *cis* and *trans* elements. The resulting landscape recapitulated HIV-1’s known *cis*-acting elements (i.e., long terminal repeat [LTR], Ψ, and Rev response element [RRE]) and, surprisingly, indicated that HIV-1’s central DNA flap (i.e., central polypurine tract [cPPT] to central termination sequence [CTS]) is as critical as the LTR, Ψ, and RRE for long-term passage. Strikingly, RanDeL-seq identified a previously unreported ∼300-bp region downstream of RRE extending to splice acceptor 7 that is equally critical for sustained viral passage. RanDeL-seq was also used to construct and screen a library of >90,000 variants of Zika virus (ZIKV). Unexpectedly, RanDeL-seq indicated that ZIKV’s *cis*-acting regions are larger than the untranscribed (UTR) termini, encompassing a large fraction of the nonstructural genes. Collectively, RanDeL-seq provides a versatile framework for generating viral deletion mutants, enabling discovery of replication mechanisms and development of novel antiviral therapeutics, particularly for emerging viral infections.

## Abstract (precis)

Recent studies have renewed interest in developing novel antiviral therapeutics and vaccines based on defective interfering particles (DIPs)—a subset of viral deletion mutants that conditionally replicate. Identifying and engineering DIPs require that viral *cis*- and *trans*-acting elements be accurately mapped.

## INTRODUCTION

A generalized feature of genome structure is the presence and interplay of *cis*-acting and *trans*-acting elements (1). In viruses, *trans*-acting elements (TAEs) comprise viral gene expression products such as proteins and RNAs that drive molecular processes involved in viral replication, maturation, and release (2). Viral *cis*-acting elements (CAEs) are sequences within the viral genome that are acted upon by TAEs or that interact with other regions of the viral genome to enable TAE-mediated genome replication, encapsidation, and other processes essential to viral maturation (3, 4). Across viral species, CAEs are conserved at the 5′ and 3′ ends, forming secondary structures such as stem loops and higher-order structures that aid genomic stability or increase interaction with TAEs (5). Function can be often inferred from location, with 5′ CAEs correlating to replication and initiation of translation, and 3′ CAEs correlating to nuclear export, RNA processing, and RNA stability (6). CAEs can also be found within gene-coding regions and function in ribosomal frameshifting, RNA replication, and specifying the RNA for encapsidation (5).

Mapping and characterization of viral CAEs have elucidated critical molecular mechanisms in the lifecycles of a number of viruses (3, 7). For example, packaging signals, frameshifting signals, and internal ribosome entry sites (IRESs) are critical CAEs and represent putative inhibition targets (8). Despite the challenges associated with disruption of structural elements, the high conservation rate of these sequences makes them attractive antiviral targets (4, 9).

One area where mapping of viral CAEs and TAEs is clearly important is in rational design of live-attenuated vaccines (LAVs) (10, 11); LAV candidates lacking CAEs have reduced replicative fitness. Thus, CAE retention may be required for efficient replication and immunogenicity of the LAV candidate. Alternatively, it is possible that deletion of CAEs could enable calibration of viral replication for attenuation. Knowledge of conserved features is also important for viruses subject to high recombination or mutation rates (12), and a rapidly implementable attenuation platform would clearly be beneficial (13). Additionally, knowledge of conserved viral regions aids the development of complementary attenuation strategies, such as microRNAs (14).

Mapping viral CAEs and TAEs may also aid development of novel classes of antivirals that act via genetic interference (15) and are proposed to have high barriers to the evolution of viral resistance. One class of such proposed antivirals are therapeutic interfering particles (TIPs), engineered molecular parasites of viruses based upon defective interfering particles (DIPs). DIPs are subgenomic deletion variants of viruses that do not self-replicate but conditionally mobilize in the presence of wild-type virus and can interfere with wild-type replication (16–23). TIPs are enhanced DIPs proposed to retain all CAEs and interfere with wild-type replication by stoichiometric competition for TAEs, such as packaging proteins, within the infected cell. Enhanced replication of DIP/TIPs, in turn, reduces the wild-type viral load. Current candidates (24–26) are generated by traditional methods of high-multiplicity of infection (MOI) serial passage or UV inactivation. A high-throughput rational genetics approach to development of DIPs and TIPs would aid screening and identification of safe and effective candidates.

Despite the benefits of mapping viral CAE and TAEs, methods to do so, especially for CAEs, tend to be laborious and/or highly technical and traditionally focus on protein-coding sequences rather than on regulatory sequences (27, 28). Highly technical methods include multicolor long-term single-cell imaging (29), CRISPR/Cas9 deletion tiling (27, 30), chemical probing approaches (31), targeted RNA mutagenesis and functional binding assays (32, 33), and bioinformatics (6, 34). Most methods, however, still rely on viral defective interfering (DI) RNAs, deducing critical genomic regions by serial passage. CAEs are, in turn, mapped by analyzing deletion variant sequences that persist or produce infectious virions. DI RNA studies reveal critical genomic regions that can be investigated further with reverse genetics systems such as site-directed mutagenesis (35–39) or iterative deletion vectors (40–44).

These approaches are limited by the ability to examine one element at a time, iterate deletions around one factor, or delete portions of the viral genome. Not all viruses have naturally occurring DI RNAs, and generating them by serial passage is straightforward but laborious. Deletion mutants arise at low frequency and remain rare unless a deletion confers increased fitness relative to the wild-type virus. A number of methods to generate defined mutants and random deletions at an appreciable frequency using reverse genetic systems exist, such as creating short random deletions with endonucleases (45) or using synthetic DNA and site-specific recombinases (46) for larger deletions. Other methods insert transposon cassettes into viral genomes to disrupt CAEs and TAEs by separating protein domains or introducing missense and nonsense mutations (47–50). These methods do not generate deletion mutants at scale, and all have certain drawbacks, whether it be nonrandom mutation/deletion, viral insertion scarring, reliance on previously characterized DIPs, inability to generate and track full-length viral mutants, or the price, labor intensity, and versatility of the method.

In this study, we present a versatile framework for generating random deletion libraries of viral species in high throughput and mapping viral CAE and TAEs without laborious and iterative deletion. Through *in vitro* transposition, dual exonuclease chewback, and barcode ligation, random deletion library sequencing (RanDeL-seq) generates diverse randomized libraries of barcoded viral deletion variants (>10^5^ unique mutants) at modest expense in fewer than 5 days. As proof of concept, we demonstrate the construction and screening of tagged libraries of >23,000 deletion mutants of HIV-1 and >90,000 deletion mutants of Zika virus (ZIKV). Repeated *in vitro* passage and deep sequencing of the pooled viral mutants comprehensively mapped HIV-1 and ZIKV at single-base resolution, identifying established viral CAEs and revealing the importance of other viral regions for sustained viral replication in cells, such as the central polypurine tract (cPPT) and splice acceptor 7 (SA-7) in HIV-1 and nonstructural proteins in ZIKV.

## RESULTS

### A method to generate a random deletion library: HIV-1 case study.

**FIG 1:**
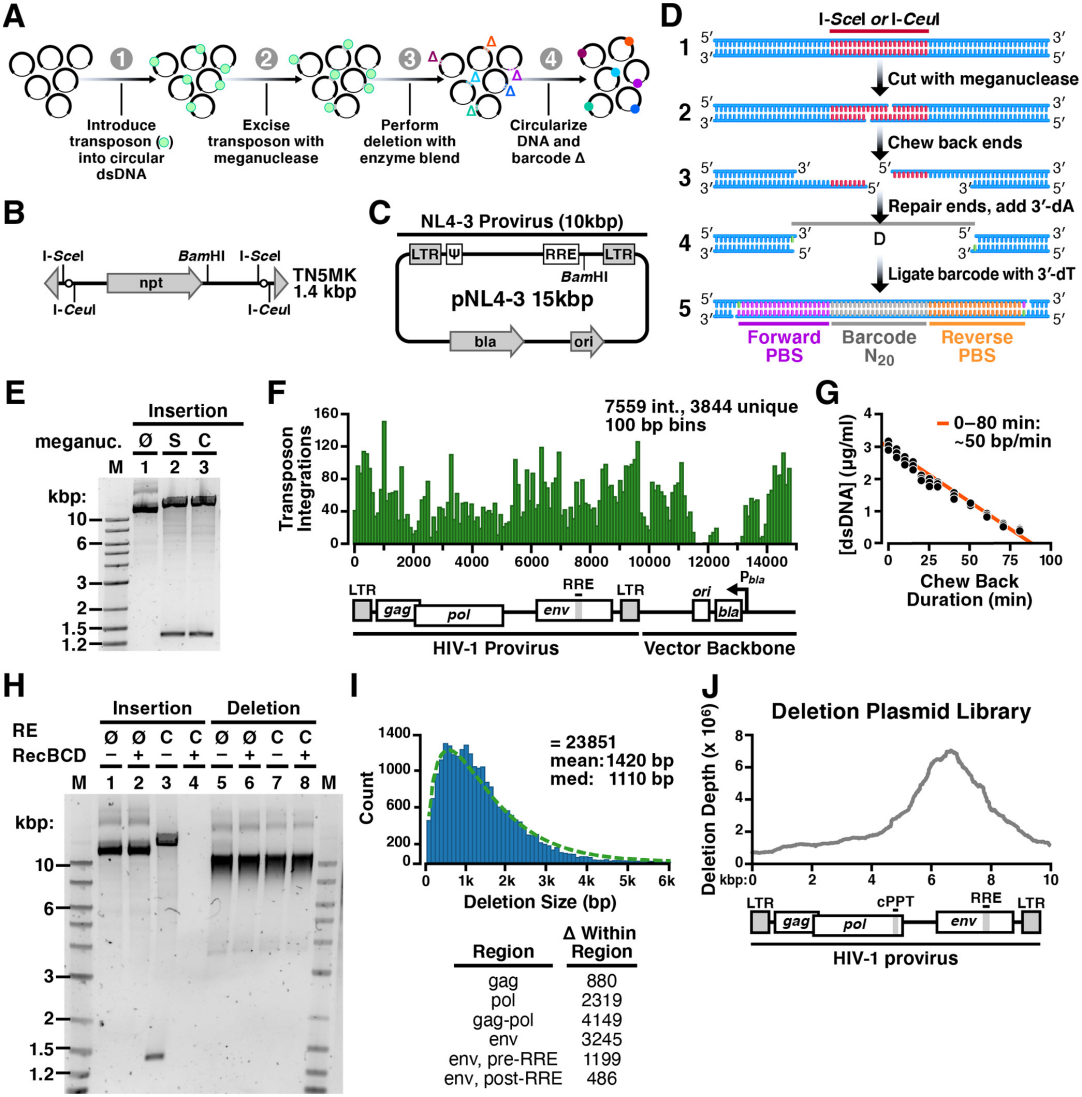
Method to generate a random deletion library in HIV-1. (A) Overview schematic of method to create a barcoded random deletion library. (1) Transposon cassettes harboring unique restriction sites are inserted into plasmids via *in vitro* transposition. (2) Transposons are excised to linearize the insertion library with a meganuclease. (3) Deletions are performed by chewback from both DNA termini by simultaneous treatment with enzyme blend. Mean deletion size is modulated by adjusting duration of chewback. (4) The chewed termini are end repaired, dA tailed, and then joined by ligation to a T-tailed 60-bp unique barcode cassette. (B) Schematic of the “TN5MK” synthetic meganuclease transposon cassette used in library construction. TN5MK is composed of an antibiotic resistance gene, neomycin phosphotransferase I (*npt*), flanked by meganuclease restriction sites for I-SceI and I-CeuI and Tn*5* mosaic ends (gray triangles) at the termini. The transposon cassette also contains a unique internal BamHI recognition site. (C) The HIV-1 molecular clone pNL4-3 is a 14,825-bp plasmid harboring the 9,709-bp NL4-3 provirus (HIV-1 subtype B). NL4-3 is a chimera of two viruses (NY5 and lymphadenopathy associated virus [LAV]). (D) Library insertion, excision, and barcoding details. (1) Circular DNA is linearized by digestion with a meganuclease (I-SceI or I-CeuI), which cleaves at recognition sites encoded on the inserted transposon. (2) This creates linear DNA with 4-base 3′ overhangs. Deletions are created by bidirectional chewback. (3) Treatment with two exonucleases (T4 and RecJ_f_) creates a population of truncated deletion mutants with ragged ends. (4) Ragged DNA ends are blunted and then prepared for barcode cassette ligation by 5′ dephosphorylation and addition of a single 3′ dA. (5) Deletion mutants are religated in the presence of a barcode cassette with single 3′ dT overhangs and 5′ phosphoryl groups to create barcoded circular DNAs with 2 nicks separated by 60 bp. Barcodes are constructed with two primer-binding sites (PBS) on either side of a unique 20-bp sequence (barcode N_20_). (E) Insertion libraries following I-SceI (S) or I-CeuI (C) digestion. Digestion of pNL4-3 insertion library shows excisions of the TN5MK transposon (1.4 kb) and upward shift of the supercoiled library versus the undigested library. Lane M, 2-log DNA ladder; 1, undigested insertion library; 2, I-SceI digested insertion library; 3, I-CeuI digested insertion library. (F) Location of TN5MK insertions for a subset of 7,559 transposon integrations (3,844 were unique). (G) Determination of enzymatic chewback rate for deletion size. The chewback rate was determined by treating a 4-kb fragment of linear dsDNA with RecJ_f_ and T4 exonucleases in the presence of SSB and no dNTPs for increasing amounts of time and then halting enzymatic activity. Reactions were performed in triplicates. DNA concentrations were established by quantifying the fluorescence of PicoGreen in a plate reader in comparison to that of a dsDNA standard of known concentration. (H) Validation of deletion library. The pNL4-3 insertion library and pNL4-3 deletion library were either not digested (∅) or cut with I-CeuI (C) and then subjected to binary treatment with RecBCD, which digests linear DNA to completion. Lanes 1 to 4 are the pNL4-3 insertion library, and lanes 5 to 8 are the pNL4-3 deletion library. (I) pNL4-3 is composed of 23,851 tagged mutants with a range of deletion sizes. The right-skewed (i.e., right-tailed) histogram of deletion sizes in pNL4-3, with bins of 100 bp (shown in blue), is well-fit by a gamma distribution (green dashed line). (Inset) Number of deletions detected within each region of the HIV genome. (J) Deletion depth profile over the full HIV-1 genome. Calculation of the deletion depth profile of the pNL4-3 genome indicates that each base is covered by hundreds to thousands of deletion mutants. Two regions where deletions are not tolerated in the plasmid backbone are ori, the origin of replication, and bla, β-lactamase, the resistance marker.

To map viral *cis*- and *trans*-acting elements, we developed RanDeL-seq, a technique to efficiently generate and screen random deletion libraries of a viral species in high throughput. The method (Fig. 1A) involves deletion via *in vitro* transposition, transposon excision, dual exonuclease chewback, and religation with molecular barcodes able to be mapped by deep sequencing. Viral mutants could be followed over time by their unique barcodes at a resolution not attainable by standard sequencing of pooled viral nucleic acids (51).

To start, we designed a synthetic transposon cassette, TN5MK (Fig. 1B), compatible with the well-characterized hyperactive Tn*5* transposase (52–54). Transposons contained an antibiotic resistance marker to select for plasmids harboring a successful transposon insertion. Transposon integration into the target plasmid introduces unique restriction sites for uncommon meganucleases, I-SceI and I-CeuI, with long recognition sites (see Fig. S1A in the supplemental material). The length of the recognition site confers specificity and is advantageous for use without modification in many systems.

10.1128/mBio.01724-20.1 FIG S1 (A) Transposon cassette: detail of meganuclease restriction sites for I-SceI and I-CeuI encoded in transposon TN5MK. (B) Minimal conditions to perform a chewback reaction. λ-HindIII digested plasmid was treated with a combination of enzymes and without dNTPs for 30 min, and then dNTPs were added to fill-in the ends at 37°C. For the various dropout reactions, dH_2_O was substituted for enzyme solutions. T4 DNA polymerase exonuclease activity is predominantly 3′ to 5′ on dsDNA when incubated with magnesium cations and no dNTPs. RecJ_f_ exonuclease activity is 5′ to 3′ on 5′ overhangs of at least 7 bp and is increased by the presence of single-stranded binding protein (SSB). The combination of the three leads to the most “smearing” on the gel, representing various shorter genome sizes. (C) Loss of BamHI sites in the deletion library pNL4-3. The pNL4-3 plasmid contains a BamHI site at base 8465, between RRE and the end of *env*. The pNL4-3 insertion library and pNL4-3 deletion library were cut with BamHI (B) and subjected to binary treatment with RecBCD, which digests linear DNA to completion. Digestion with BamHI shows a smear in the insertion library (lane 9), where the sizes differ depending on where the TN5MK integrates relative to the BamHI site. Treatment with RecBCD shows no DNA remaining (lane 10). However, in the deletion library, we see a range of sizes peaking around 15 kbp (lane 11), and a population is resistant to BamHI/RecBCD (lane 12), indicating deletion of the preexisting BamHI site. Download FIG S1, TIF file, 2.8 MB. Copyright © 2021 Notton et al. 2021 Notton et al. This content is distributed under the terms of the Creative Commons Attribution 4.0 International license.

The conventional HIV-1 molecular clone pNL4-3 (Fig. 1C) was the substrate for this library construction. The system allows control over the size of deletions and can tag each member of the diverse deletion library with a molecular barcode to facilitate deep-sequencing analysis. The molecular biology details of RanDeL-seq are in Fig. 1D. Each step was validated after completion, with a comprehensive check on the finished libraries.

First, we performed *in vitro* transposition to randomly insert TN5MK into pNL4-3 at a ratio of one transposon per viral plasmid. The insertion libraries were treated with both encoded restriction enzymes, generating the expected ∼1.4-kb excised transposons in addition to linearized pNL4-3 plasmid backbone (Fig. 1E). Deep sequencing of the pNL4-3 insertion library enabled mapping of insertion sites across the genome (Fig. 1F), showing that TN5MK integrated broadly throughout the pNL4-3 plasmid, with a high frequency and density of at least one transposon integrated every 100 bp. In the plasmid backbone, two integration gaps emerged, one in the origin of replication (ori) and the other in the resistance marker, as both are required for propagation of the plasmid in Escherichia coli.

After creating a polyclonal population of transposon-inserted circular target DNAs, insertions were excised by meganuclease treatment. DNA chewback with a trio of proteins (T4 DNA polymerase, RecJ_F_, and single-stranded binding protein [SSB]) efficiently created truncations in a common buffer system (Fig. S1B). Chewback rate was determined by a double-stranded DNA (dsDNA) fluorometric method, using a 4-kb template DNA. As the ends were progressively shortened by chewback, the fluorescence signal of a dsDNA-specific dye (PicoGreen) decreased proportionally. The measured double-end chewback rate, as determined by linear regression, was approximately 50 to 60 bp/min (Fig. 1G). Sublibraries of mutants with diverse deletion sizes were then created by varying the enzymatic incubation time. Finally, linearized sublibraries were pooled, end repaired, deoxyribosyladenine (dA) tailed, dephosphorylated, and recircularized by ligation to a 3′ deoxyribosylthymine (dT)-tailed 60-bp barcode cassette. The barcode cassette was designed to have a 20-bp random barcode flanked by 20-bp primer-binding sites, taken from Tobacco mosaic virus to limit sequence complementarity with human viruses (see Fig. S2). Each successful ligation resulted in a deletion mutant tagged with a unique barcode cassette.

10.1128/mBio.01724-20.2 FIG S2 Sequences of transposon and barcode cassettes (HIV and ZIKV). (A) Detail of HIV barcode cassette with left and right common sequences flanking a unique 20-nt barcode. (B) Detail of ZIKV barcode cassette with left and right common sequences flanking a unique 20-nt barcode. Substitution made from HIV barcode cassette to replace triple Gs. Download FIG S2, TIF file, 2.8 MB. Copyright © 2021 Notton et al. 2021 Notton et al. This content is distributed under the terms of the Creative Commons Attribution 4.0 International license.

We validated the final library via several different methods (Fig. 1H). First, to test if the transposon insertion was fully excised, libraries were restriction enzyme digested by I-CeuI. The completed library (Fig. 1H, lane 7) was insensitive compared to a digested insertion library (lane 3), confirming TN5MK excision and removal in chewback. Second, an untreated deletion library had a range of subgenomic sizes (Fig. 1H, lane 5) in comparison to an untreated insertion library (lane 1), confirming that chewback created deletions of various sizes. Lastly, to test successful recircularization with the barcode cassette, the digested insertion and deletion libraries were treated with RecBCD, an enzyme that degrades linear dsDNA. We hypothesized that treated insertion libraries and deletion libraries that maintained I-CeuI cut sites or were not properly ligated to barcode cassettes would be completely degraded. Posttreatment, the deletion library was unchanged, as the digested plasmids were uncut and circular from ligation to the barcode cassette (Fig. 1H, lanes 6 and 8). On the other hand, treatment of insertion libraries degraded all plasmid (Fig. 1H, lane 4). RecBCD treatment of insertion and deletion libraries digested with BamHI, an internal site in HIV, further validated these results (Fig. S1C).

### Framework for efficiently sequencing barcoded HIV-1 RanDeL.

Postvalidation of the tagged RanDeL, we developed a framework for genotyping barcoded mutants in order to track each unique deletion mutant in culture and calculate a viral deletion depth profile. RanDeL-seq relies on the initial whole-genome sequencing of the deletion library to construct a lookup table that links each unique barcode sequence to a specific deletion locus. This initial genotyping step allows for efficient sequencing downstream, as only barcode cassettes need to be sequenced downstream to identify which deletion mutants persist in culture.

The plasmid library was fragmented, deep sequenced (2 × 125-bp reads on HiSeq 4000) with Illumina paired-end sequencing and analyzed with custom python software (rdl-seq). Reads were filtered for the small percentage (2.9%) that contained the full barcode cassette (see Table S1). Repeated barcode sequences were grouped together to determine the consensus bases 5′ and 3′ of that specific barcode cassette (i.e., barcode-flanking sequences). Flanking sequences were aligned to the viral reference genome, generating a lookup table of barcodes (B = b_1_, b_2_, b_3_, …, b_n_) matched to deletion loci (D = d_1_, d_2_, d_3_, …, d_n_). After the initial genotyping of the plasmid random deletion library, deletion variants can be identified by amplifying barcode cassettes with primers annealing to the common primer-binding sites.

10.1128/mBio.01724-20.9 TABLE S1 Summaries of HIV and ZIKA plasmid RanDeL whole-genome sequencing. Download Table S1, DOCX file, 0.4 MB. Copyright © 2021 Notton et al. 2021 Notton et al. This content is distributed under the terms of the Creative Commons Attribution 4.0 International license.

This sequencing framework determined there were 23,851 unique mutants with a range of deletion sizes (Fig. 1I). The library subset had a median deletion size of ∼1.1 kb, a minimum deletion of 30 bp, and a maximum deletion size of >6 kb. The skewing of the library (i.e., long tail toward large kilobase sizes) may be due to mechanical shearing of DNA during some of the cleanup steps.

Next, the deletion depth profile (location and abundance of deletions) of the pNL4-3 deletion library was calculated (Fig. 1J). The plasmid library exhibits deletions across the HIV genome, with a peak at the *env* gene, and a region of zero deletion depth was observed at the plasmid ori and antibiotic resistance marker (bla). Biases in the deletion depth at this stage correspond to differences in bacterial growth rate; faster growing bacteria lead to overrepresentation of their harbored plasmid. The signal peptide of *env* and sequences at the N terminus are known to be toxic to bacteria (55); therefore, bacteria harboring *env* deletions likely have a growth advantage, and bacteria harboring ori/bla deletion plasmids are unable to grow in the antibiotic.

### Serial-passage screening of HIV-1 RanDeL to map viral CAEs and TAEs.

To functionally characterize the deletion library, we designed a high-multiplicity of infection (MOI) passage scheme to select for and map viral CAEs by sequencing the barcodes that persisted through multiple passages together with replication-competent HIV-1. A high MOI ensured that on average, each cell became infected with more than one copy of the wild-type virus to supply *trans* factors. The diversity of the library was limited to fewer than the number of available cells to maintain strong selective pressure, avoid drift, and ensure that most of the library would be sampled multiple times during infection. In this scheme, the genomic regions that can tolerate deletion (as measured by enrichment of specific barcodes corresponding to that region) correspond to TAEs, while regions that are intolerant of deletion correspond to CAEs.

**FIG 2:**
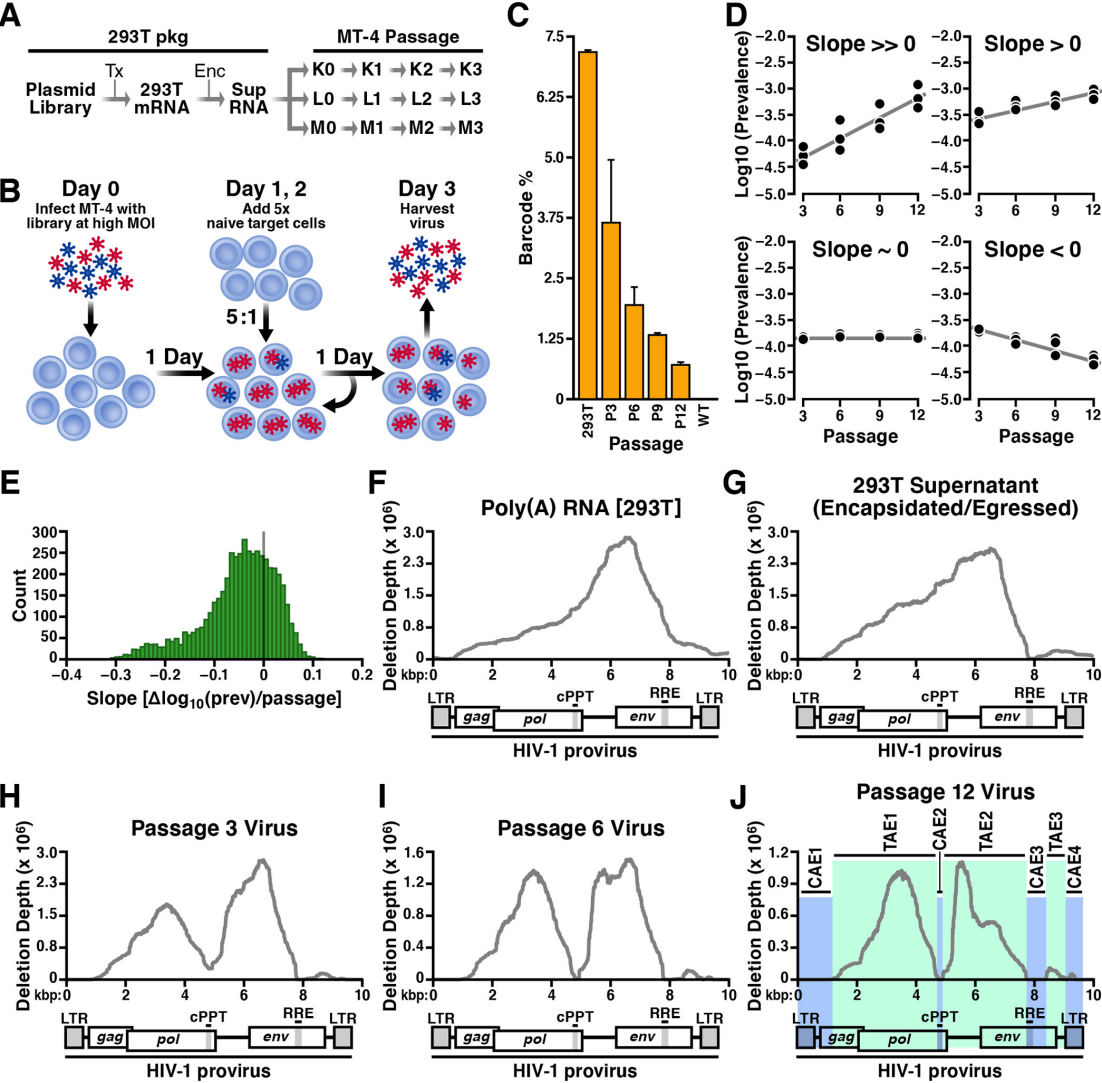
Genetic screen of random deletion library to map viral *cis* and *trans* elements. (A) Block design of high-MOI passage. Wild-type NL4-3 and deletion library plasmids cotransfected 293T cells. Virus-containing supernatant-infected MT-4 cells in triplicates (K, L, M) at high MOI. Supernatant was collected and transferred onto new cells at the end of every week, for 4 weeks. At the same time, flasks with only NL4-3 wild-type virus were passaged identically. (B) Passage details of high-MOI screen. MT-4 cells (blue double discs) are infected at high MOI with a pool of virus (HIV-1) containing both wild-type (red stars) and deletion mutants (blue stars). At days 1 and 2, additional naive MT-4 cells were added and the culture volume expanded. On day 3, cell-free supernatant was harvested and virus purified by ultracentrifugation for transfer or analysis. There were 3 total rounds of HIV replication from day 0 to day 3. (C) Detection and quantification of barcode cassettes by RT-qPCR. Genomic percentages of barcoded mutants to total HIV genomes in transfection (293T), each stage of high-MOI passage (P3 to P12), and a wild-type HIV control (WT). RT-qPCR data were normalized to an MS2 RNA spike-in. Error bars are standard deviations from averaging each flask (K, L, and M) per passage. (D) Representative deletion variant trajectories during high-MOI passage. The slope in prevalence versus passage number was determined by linear regression. Data points correspond to the triplicate flasks (K, L, and M) at each passage. Prevalence is in reference to the total barcode cassette pool (tagged mutants). (E) Distribution of fitness in deletion variants that are not extinct by passage 12. The vertical line marks a slope of 0 for reference. (F) The deletion depth profile of poly(A) RNA from transfected 293T cells, representing mutants able to be transcribed. (G) The deletion depth profile built from the virus-containing supernatant of transfected 293T representing mutants able to be transcribed, encapsulated, and egressed. (H) Deletion depth profile from virus-containing supernatant after 3 passages. (I) Deletion depth profile after 6 passages. (J) A model of HIV-1 *cis*- and *trans*-acting elements after 12 passages. The HIV-1 genome is composed of 4 *cis*-acting elements, CAE1 to CAE4 (highlighted in blue), and 3 *trans*-acting elements, TAE1 to TAE3 (highlighted in green).

Wild-type virus and deletion library pools were packaged by cotransfection of 293T cells with equal masses of the pNL4-3 deletion library and pNL4-3 parental plasmid. Clarified supernatant (0.45-μm filtered) was concentrated by ultracentrifugation and used to transduce MT-4 cells at high MOI in three parallel biological replicates (designated K, L, and M) for 12 passages (Fig. 2A). In parallel, three flasks were infected with wild-type HIV-1 only as a negative control for deletion library barcodes. In this high-MOI passage scheme (Fig. 2B), cultures were infected with concentrated virus and then supplemented with naive MT-4s every 24 h before being harvested 3 days (i.e., 3 passages) later. By supplying naive target cells, the scheme selected for two phenotypes: (i) replication-competent viruses and (ii) replication-defective viruses that are efficiently *trans*-complemented by wild-type virus (i.e., mobilized). Flow cytometry under high-MOI conditions showed an initial high percentage of infected cells, followed by an expected drop after the addition of naive MT-4s and then a return to high infected percentage before harvest (see Fig. S3).

10.1128/mBio.01724-20.3 FIG S3 High MOI is maintained throughout each stage of a week of passage: after infection with concentrated virus, after addition of naive MT-4 target cells, and at harvest of virus. Cells that were positive for HIV-1 capsid protein are indicated in the polygonal gate and reported as a percentage of the population. For each sample, the largest flow cytometry dot plots depict side scatter (SSC) versus enhanced green fluorescent protein (EGFP), and the gating used to establish which cells are EGFP positive; the small upper right plots depict live cell gating (forward scatter [FSC] versus SSC), and the small lower right plots show singlet gating (forward scatter width versus forward scatter area). Download FIG S3, TIF file, 2.8 MB. Copyright © 2021 Notton et al. 2021 Notton et al. This content is distributed under the terms of the Creative Commons Attribution 4.0 International license.

### Tracking RanDeL barcodes throughout serial-passage experiments.

Viral RNA from cell-free supernatants was analyzed by reverse transcription-quantitative PCR (RT-qPCR) to detect barcode sequences and determine which deletion variants persisted passage to passage. Barcodes were detectable in all deletion library samples in the serial-passage flasks and in none of the control flasks. The ratio of barcodes to total HIV genomes slightly decreased over time from the initial cotransfection of deletion library samples (Fig. 2C). Expression of total HIV genomes was not significantly different between the library and control samples (see Fig. S4A), indicating no interference from the deletion library.

10.1128/mBio.01724-20.4 FIG S4 (A) High-MOI passage quantification: fold change in total HIV genomes from HIV-1 RanDeL library compared to RanDeL control. Total HIV genomes were calculated by RT-qPCR of viral RNA using primers for the POL region. Fold change is measured at each stage of high-MOI passage. (B) HIV biological replicates show strong concordance between triplicate infections. Pairwise correlation plots of all barcodes in triplicates at passages 3 and 12 show *R*^2^ values of 0.83 to 0.93 at passage 3 and passage 12. Each axis is the log barcode prevalence within an individual flask. Download FIG S4, TIF file, 2.8 MB. Copyright © 2021 Notton et al. 2021 Notton et al. This content is distributed under the terms of the Creative Commons Attribution 4.0 International license.

Using a custom Illumina-prep library for barcode sequencing (see Fig. S5), the prevalence of each deletion variant was calculated by dividing the total sequenced copies of a particular barcode by total sequenced barcodes. Of the 23,851 mappable pNL4-3 deletion mutants, only 4,390 (18%) were detectable in all three replicate flasks by passage 12—the remaining 19,461 (82%) barcodes were undetectable and presumably were extinct in at least one of the three replicates. Overall, there was strong concordance in barcode prevalence between the three replicates (Fig. S4B).

10.1128/mBio.01724-20.5 FIG S5 Preparation of Illumina sequencing libraries from PCR of barcode cassettes, version 1. Three consecutive PCR reactions were used to add adapter sequences compatible with the TruSeq sequencing system. Introduction of molecular barcodes (orange) and multiplexing barcodes (green) allowed for high-throughput quantification of the barcoded libraries. Download FIG S5, TIF file, 2.8 MB. Copyright © 2021 Notton et al. 2021 Notton et al. This content is distributed under the terms of the Creative Commons Attribution 4.0 International license.

For the 4,390 persistent barcoded deletions, we calculated the change in prevalence versus passage number (i.e., slope) by linear regression and classified variants by slope (Fig. 2D). Linear regression analysis determined that 1,390 (32%) of the 4,390 persisting deletion variants increased in prevalence through every passage, indicating that variants harboring these deletions transmitted faster than the average member of the barcoded population (Fig. 2E). The remaining 3,000 mutants remained steady or decreased in prevalence passage to passage. We note that the vast majority of mutants in Fig. 2E have a negative slope, in line with classical theory that most mutations are deleterious (56, 57). It is plausible that the 1,390 persisting variants are transmissible and can be efficiently complemented in *trans* (i.e., their deletions can be compensated for by gene products expressed from wild-type HIV-1). Mutants with a slope of 0, and even some mutants with a slope <0, are not necessarily deleterious, since a particular mutant’s classification depends upon the overall population trajectory of barcodes.

### HIV-1 deletion landscapes identify CAEs and TAEs.

Using deep-sequencing counts of barcodes and referencing back to the barcode-to-genotype lookup table, deletion landscapes (also known as deletion depth profiles) were calculated for the HIV-1 genome at various time points in the screen. First, we sequenced barcodes in intracellular poly(A) RNA purified from the 293T cells (Fig. 2F) used to package the deletion library. The 5′ end of the HIV-1 genome (spanning the 5′ long terminal repeat [LTR] through SL1 to SL4) exhibited low deletion depth, while the rest of the genome showed little reduction in barcode coverage. This deletion landscape reflects known CAEs required for efficient HIV-1 transcription in 293T cells.

Next, barcodes were sequenced from the cell-free supernatant of 293T cells (Fig. 2G), representing deletion variants able to be transcribed, packaged into virions (encapsidated), and released from the cell (egressed). This supernatant deletion landscape differed from the intracellular RNA deletion landscape in two key genomic regions: (i) the region of zero deletion depth beginning at the 5′ LTR and extending through the start codon of *gag*, which includes the HIV-1 packaging signal (*psi* [Ψ]), and (ii) at the 3′ end of the genome, the stretch of zero deletion depth that maps to the Rev responsive element (RRE), a region of secondary structure critical for nuclear export of incompletely spliced HIV-1 RNAs (58, 59). These data indicate that the LTR, Ψ, and RRE were the only elements critical for efficient transcription, encapsidation, and egress of HIV-1 from 293T cells; all other regions tolerated some amount of deletion.

Deletion landscapes were then calculated to profile the changes in the deletion library passage to passage in MT-4 cells. At passage 3 (Fig. 2H), the deletion landscape diverged notably from the 293T-intracellular and supernatant profiles in three key ways: (i) a valley of reduced deletion depth appeared, with a minimum centered above the cPPT/central termination sequence (CTS), (ii) the region of zero deletion depth at the 5′ end of the genome shifted, encompassing the 5′ LTR through the first 300 bases of *gag*, and (iii) a widening and 3′ shift of the deletion depth valley situated around the RRE occurred. At passage 6 (Fig. 2I), these features had become more pronounced, and each valley had flattened to a deletion depth near zero.

No significant landscape differences were found between passages 9 and 12, enabling construction of a consensus map (Fig. 2J). Three regions of the HIV-1 genome were tolerant to deletion and able to be complemented efficiently in *trans*. These deletion-tolerant regions were classified as TAEs and are (i) a region centered at the deletion peak at the center of *pol* (TAE1), (ii) a region in HIV’s accessory gene tract (*vif-vpu*) (TAE2), and (iii) a region in the 3′ end of *env* (TAE3).

The final deletion depth profile contained four regions of low or zero deletion depth, indicating that these genomic regions are required CAEs. CAE1 is the first 1,114 nucleotides of the proviral genome, encompassing known CAEs, the 5′ LTR, stem loops 1 to 4, and the first 325 bp of *gag*, which maps to the Gag MA (p17) and Ψ. CAE2 maps directly to the cPPT/CTS. The requirement for HIV cPPT in reverse transcription and integration has been debated in the past, with the literature variously supporting (31, 60–64) and questioning (65–68) its role. Here, the data support a critical role for cPPT in sustained HIV-1 replication. CAE3 begins at the RRE and ends precisely at splice acceptor 7 (SA-7), which is used for several multiply spliced HIV-1 transcripts, including *vpr*, *tat*, *rev*, and *nef* (69), and is implicated in viral fitness (59). While the importance of the RRE and SA-7 were known (31, 70, 71), RanDeL-seq showed that the entire 300-bp region from the upstream RRE to the end of SA-7 is required for sustained viral replication and cannot be provided in *trans*. Finally, CAE4 spans the PPT, which is necessary for reverse transcription, and the 3′ LTR (18).

### Validation of *trans*-acting regions via traditional methods.

**FIG 3:**
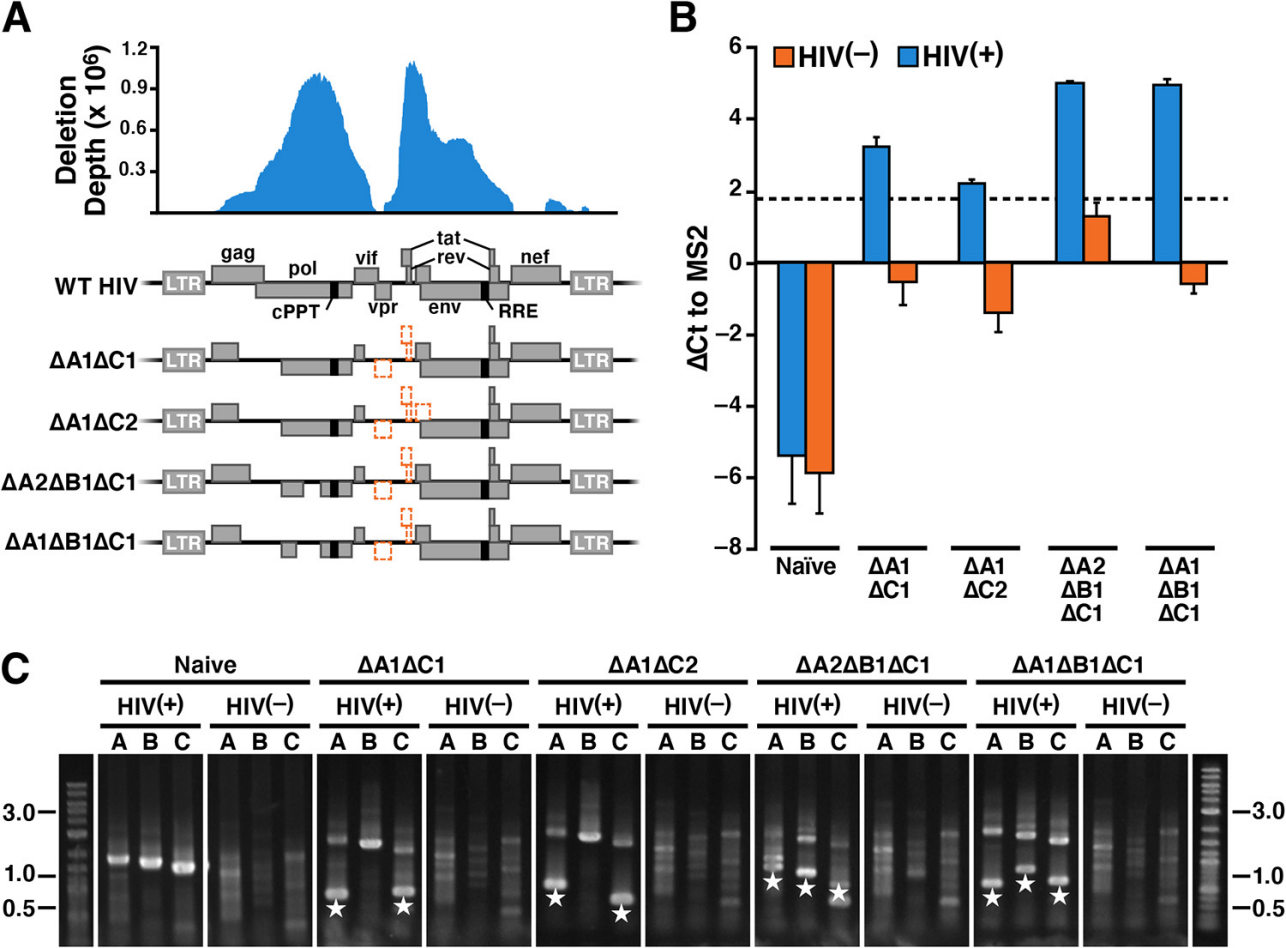
Validation of *trans*-acting regions. (A) Schematics of recombinant HIV-1 mutants with deletions in *trans*-acting regions. The HIV-1 deletion depth profile (from Fig. 2) is shown over the genomic schematic of HIV-1 (WT HIV), with major coding sequences and proteins labeled. Schematics for the four recombinant deletion vectors are at the bottom, with names corresponding to deleted loci (deleted regions are shown as orange dashed regions). There are two different “A” deletions (ΔA1 and ΔA2) focused in the *gag/pol trans*-element, a “B” deletion focused in *pol* (ΔB1), and two “C” deletions in the HIV accessory proteins and the beginning of *env* (ΔC1 and ΔC2). The mapped *cis*-acting elements, cPPT and RRE/SA-7, are colored black in the schematic. The deletion coordinates by nucleotide location are A1 (1455 to 2815), A2 (1636 to 2651), B1 (3620 to 4606), C1 (5073 to 6058), and C2 (5041 to 6251). (B) Mobilization of recombinant deletion vectors occurs only in the presence of HIV-1 superinfection; RT-qPCR analysis of barcodes in cellular supernatant. Δ*C_T_* values are relative to an MS2 spike-in control. Positive Δ*C_T_* values for barcode in HIV(−) samples are likely due to barcode primer dimers. The limit of detection due to primer dimers was determined to be ∼1.3 (dashed line). Error bars are standard deviations from technical replicates. (C) Transmission of recombinant deletion mutants to new target cells occurs only in the presence of HIV-1 superinfection; recombinant deletion mutants are detected in cellular genomic DNA by PCR following supernatant transfer in the presence of HIV-1 infection. The deleted regions are detected by PCR using primers spanning the deleted regions A, B, and C (i.e., the lanes A, B, and C in each gel). In naive cells (no deletion vector), the PCR products are detected at the expected sizes, 1.6 to 1.9 kb each, only in the presence of HIV-1. In cells carrying the recombinant deletion vectors, the PCR products are detected at the expected sizes of ∼0.5 kb only in the presence of HIV-1. Mutant deletion blocks are marked with white stars.

To validate the RanDeL-seq approach, we used traditional recombinant lentiviral vector cloning approaches to test if regions of HIV-1 found to be *trans*-acting could in fact be complemented in *trans*. Four recombinant HIV-1 deletion variants with deletions in TAE1 and TAE2 were cloned (ΔA1ΔC1, ΔA1ΔC2, ΔA2ΔB1ΔC1, and ΔA1ΔB1ΔC1) (Fig. 3A). Deletions were chosen based on the calculated CAE and TAE map and were concentrated in *gag*, *pol*, and the accessory proteins (*vif*, *vpr*, *tat*, *rev*, and *vpu)*. Each deletion region corresponded to a letter (A, B, or C) for naming purposes. The sequence between deletions A and B was included to aid vector cloning and is not anticipated to have any extra effect due to a frameshift in *pol*. Each mutant encoded an identical RanDeL-seq barcode for qPCR detection and quantification.

To test if the cloned vectors could be *trans*-complemented, we designed a mobilization assay to determine if the deletion vector could replicate and transmit in the presence or absence of HIV-1. HIV deletions were packaged by standard methods and used to transduce MT-4 cells. Four days postransduction, cells were superinfected with either HIV-1 or a medium negative control, and 2 days later, supernatant viral RNA was isolated and quantified by RT-qPCR.

The barcodes for all four deletion vectors were detected in the supernatant viral RNA (Fig. 3B) postsuperinfection with HIV-1, as measured by the change in threshold cycle (Δ*C_T_*) to an MS2 spike-in control. Barcode values were above the limit of detection for all HIV(−) samples (Δ*C_T_*, ∼1.3 due to barcode primer dimers). Therefore, the deletion vectors were not able to self-replicate and were only able to mobilize from MT-4 cells in the presence of wild-type virus.

Next, to confirm if the deletion vectors were able to transmit and integrate, we performed supernatant transfer assays. We created cell lines carrying each deletion variant, superinfected each line with HIV-1 or a negative control, transferred supernatant to naive MT-4 target cells, and isolated genomic DNA from the target cells to determine if the deletion vectors integrated. PCR analysis of each mutant block (A, B, and C) showed that the deletion vectors were only detected in HIV(+) samples (Fig. 3C). These data demonstrate that elements identified by RanDeL-seq as *trans* are *trans*-complemented by wild-type virus and do transmit.

### Application of RanDeL-seq to identify Zika virus CAEs.

**FIG 4:**
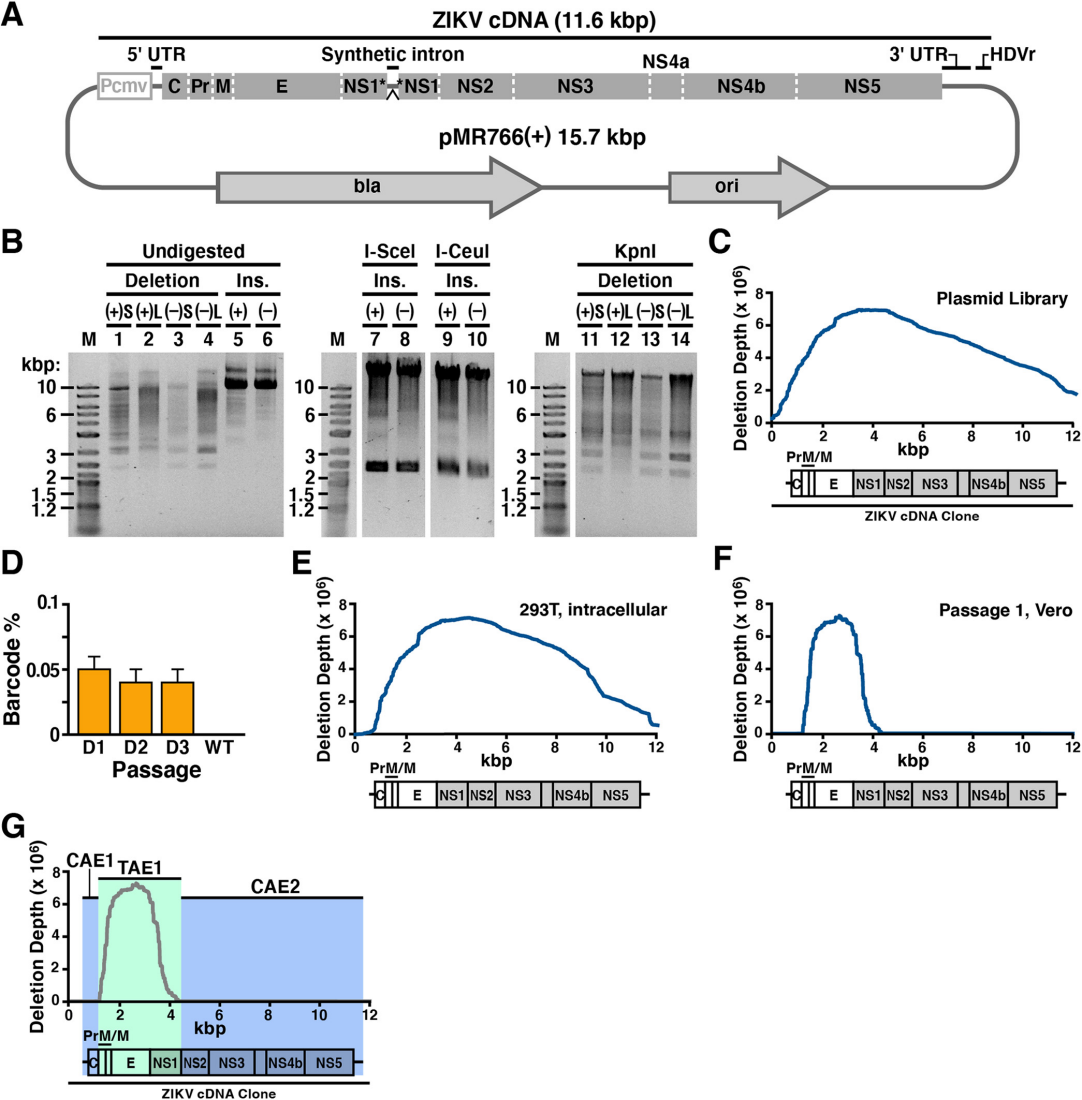
Application of RanDeL-seq to map Zika virus (ZIKV) *cis* elements. (A) pMR766(+), a Zika virus molecular clone. The MR766 Zika virus genome is encoded as a cDNA driven by the CMV IE2 promoter. At the 3′ end of the genome, a self-cleaving hepatitis delta virus ribozyme allows for creation of an authentic 3′ end posttranscription. An intron sequence is present within NS1 to allow maintenance in bacteria but is spliced out during transcription in host cells. (B) Restriction enzyme characterization of completed ZIKV deletion libraries compared to insertion libraries (“Ins.”). (+) and (−) designate the template ZIKV plasmid. “S” and “L” designate the chewback length for deletion libraries. Undigested completed deletion libraries (lanes 1 to 4) were run next to undigested insertion libraries (lanes 5 and 6). Insertion libraries (lanes 7 to 10) treated with I-SceI or I-CeuI to excise transposon (∼1.4 kb). Deletion libraries linearized by unique ZIKV cutter KpnI (lanes 11 to 14). (C) Deletion depth profile of the pMR766(+)L library. The ZIKV genome is well represented in the pMR766(+)L library, with some bias. Each base of the ZIKV genome is covered by several hundred different deletion mutants. (D) Detection and quantification of ZIKV barcode cassettes by RT-qPCR. Genomic percentages of barcoded mutants to total ZIKV genomes at each day in passage 1 of the high-MOI screen and a wild-type ZIKV control (WT). RT-qPCR data were normalized to an MS2 RNA spike-in. (E) Deletion depth profile of intracellular RNA of 293T cotransfected with the wild-type ZIKV plasmid and the pooled deletion libraries. (F) Deletion depth profile of pMR766(+)L after passage 1. Only deletions in Pr to NS1 can be *trans*-complemented by wild-type ZIKV. (G) Final map of ZIKV *cis*- and *trans*-acting elements after passage 2. The two *cis*-acting regions are highlighted in blue and do not tolerate deletion (i.e., must be present for efficient transmission to occur). The *trans*-acting region is highlighted in green and can be complemented in *trans* (i.e., if deleted, transmission occurs by complementation from wild-type virus).

To determine if this approach has the potential to be more generally applicable across diverse viruses, we performed RanDeL-seq on Zika virus (ZIKV). ZIKV is a flavivirus with a (+)-stranded, single-stranded RNA (ssRNA) genome of approximately 11 kb that replicates predominantly in the cytoplasm of infected cells. Libraries were built using two cDNA molecular clones of the conventional 1947 Ugandan strain of ZIKV, MR-766 (72). The first clone, Pol(+) pMR766, encodes the wild-type virus (Fig. 4A), whereas the second clone, Pol(−) pMR766, encodes a defective mutant with a substitution in the active site of the essential RNA-dependent RNA polymerase NS5. Consequently, pMR766(−) virus is not replication competent unless rescued by providing NS5 in *trans*. pMR766(+) and pMR766(−) insertion libraries were generated with TN5MK. Next, the transposon was excised, enzymatic chewback was performed to generate deletions, and the cDNA was recircularized by ligation in the presence of a random barcode cassette. Both short (S)- and long (L)-duration chewbacks were performed for each insertion library to create small and large average deletion sizes, respectively. Overall, four ZIKV RanDeLs were generated: pMR766(+)S, pMR766(+)L, pMR766(–)S, and pMR766(–)L.

Each ZIKV RanDeL was validated per methods similar to those used for the HIV-1 library (Fig. 4B). Restriction enzyme analysis with I-SceI and I-CeuI showed transposon excision in both sets (+ and −) of insertion libraries (Fig. 4B, lanes 7 to 10). Undigested completed deletion libraries ran at 2 to 10 kb (Fig. 4B, lanes 1 to 4), confirming various-sized deletions as a result of chewback incubations. Successful plasmid religation and recircularization of each deletion library was analyzed by restriction analysis with KpnI, a restriction enzyme with a single unique site in both ZIKV wild-type plasmids. Consistent with successful plasmid recircularization, KpnI digestion generated single bands (Fig. 4B, lanes 11 to 14), i.e., linearized molecules arising from a cut of a circular plasmid as opposed to two molecules arising from cutting of a noncircularized linear DNA molecule. These KpnI-digested single bands migrated at sizes larger than the undigested supercoiled libraries (Fig. 4B, lanes 1 to 4).

Whole-plasmid sequencing of the four ZIKV RanDeLs determined the deletion diversity to be between 1,000 and 50,000 mappable deletions per library (Table S1). Short-chewback libraries had less diversity than long-chewback libraries, likely due to some short-chewback reactions failing to chew past the transposon cassette, rendering it impossible to determine the mutation location. The deletion size distribution of the ZIKV RanDeLs differed from that of the HIV-1 RanDeLs (see Fig. S6), in that ZIKV RanDeL distributions were clearly bimodal, with peaks at small and large deletion sizes. Increasing the length of the chewback shifted the lower peak, but not the upper peak, possibly indicating that clones that lost the ZIKV cDNA insert had a replication advantage in bacteria.

10.1128/mBio.01724-20.6 FIG S6 ZIKV RanDeL deletion size distributions. pMR766 deletion libraries have a bimodal distribution of deletion sizes. Histograms of deletion sizes are shown for the four ZIKV deletion libraries: two short-chewback, pMR766(−)S (A) and pMR766(+)S (B), and two long-chewback, pMR766(−)L (C) and pMR766(+)L (D). Download FIG S6, TIF file, 2.8 MB. Copyright © 2021 Notton et al. 2021 Notton et al. This content is distributed under the terms of the Creative Commons Attribution 4.0 International license.

The pMR766(+)L plasmid library showed deletions across the ZIKV genome (Fig. 4C), with a peak at NS1 and a region of zero deletion depth at the flanking region of the genome, corresponding to the plasmid backbone (ori/bla). Given that deletion variants from the pMR766(+)S, pMR766(–)L, and pMR766(–)S libraries did not ultimately passage efficiently in cells, we did not construct deletion landscapes for the libraries from these plasmids. Similar to the HIV plasmid landscape, deletion of these backbone regions compromised the ability of the plasmid to be propagated effectively in bacteria. The peak centered at NS1 reflected increased growth of mutants with deletion in NS1, which is known to have cryptic promoter activity and cause reduced growth in E. coli (63, 72, 73).

As with HIV-1, wild-type ZIKV and RanDeL variants were packaged by cotransfection of 293T cells using equal masses of each ZIKV RanDeL and the wild-type clone. Filtered, concentrated virus-containing supernatant was isolated, pooled, and used to infect Vero cells at high MOI (>16). The viral pool was passaged three times in Vero cells in parallel to a wild-type-only control infection.

Viral RNA was analyzed from transfected 293T cells and cell-free supernatant at each passage by RT-qPCR to quantify RanDeL barcodes and total ZIKV genomes. First, we verified that barcoded mutants could be detected intracellularly posttransfection of each individual library (see Fig. S7A) and each day postinfection (dpi) in passage 1 (Fig. 4D). At 1 dpi, barcodes represented <0.01% of total Zika genomes and did not increase in percentage by 3 dpi. Total viral genomes (ZIKV capsid protein [ZIK-C]) in RanDeL cotransfected samples were not significantly different from those with the control infection (Fig. S7B), indicating the absence of a detectable interference effect from ZIKV variants, in agreement with the HIV results. However, a significant drop-off in barcode prevalence was observed between intracellular RNA posttransfection and supernatant RNA postinfection, indicating a strong selective pressure (i.e., bottleneck) on RanDeL variants between transcription and egress.

10.1128/mBio.01724-20.7 FIG S7 qPCR data of ZIKV passage. (A) Percentages of barcodes out of total ZIKV genomes in intracellular RNA from each deletion library cotransfection. Ratio of barcodes to ZIK-C. (B) Fold changes in total ZIKV genomes from ZIKV RanDeL to ZIKV control. Fold change is measured at each stage of high-MOI passage. Download FIG S7, TIF file, 2.8 MB. Copyright © 2021 Notton et al. 2021 Notton et al. This content is distributed under the terms of the Creative Commons Attribution 4.0 International license.

To identify CAEs, deletion depth profiles were constructed by Illumina sequencing of ZIKV RanDeL barcodes after cotransfection, passage 1, and passage 2. The deletion landscape of intracellular RNA in cotransfected 293T cells was similar to the plasmid profile with a couple of notable exceptions (Fig. 4E). First, at the 5′ end of the genome, deletions of the internal cytomegalovirus (CMV) promoter (bases 1 to 721) inhibited transcription, because the CMV promoter is required for transcription of the ZIKV RNA genome. Second, at the 3′ end of the genome, deletions of the hepatitis delta virus (HDV) ribozyme and poly(A) sequence also inhibited transcription.

Next, we analyzed deletion landscapes from the serial passage in Vero cells. Although the pool of viral deletion mutants for infection was initially all four sublibraries, less than 1% of detectable barcodes were from pMR766(−) libraries by the end of passage 2. The vast number of the observed barcodes (≈95%) were derived from the pMR766(+)L library, with the remaining barcodes (≈5%) from the pMR766(+)S library. One potential reason is that pMR766(−) genome replication was unable to be rescued by the wild-type virus supplying NS5 in *trans*.

Of the initial 40,000 mappable barcodes of pMR766(+)L, only 300 were detected after passage 1. These were used to construct a deletion profile (Fig. 4F), which shows a single peak, centered at *E*, that slopes downward in each direction to a deletion depth of zero beyond the 5′ border of *PrM* and the 3′ border of *NS1*. Importantly, Pr, M, and E are 3 of the 4 structural proteins that comprise the viral particle (C is the last). By passage 2, several variants with 1- to 2-kb deletions that spanned this region increased 200 to 500× in prevalence. Flavivirus replicon systems have previously been developed by deletions in these regions, including C (41, 74). RanDeL-seq determined that ZIKV tolerated deletions in Pr, M, and E but not in C.

A final deletion landscape of ZIKV by passage 2 (Fig. 4G) showed that only a 3-kb genomic interval of pMR766 can tolerate deletion. The region beginning exactly at Pr and ending precisely at the end of NS1 is TAE1 and can be efficiently complemented in *trans*. The regions flanking TAE1 are *cis*-acting, with CAE1 encompassing the 5′ untranscribed region (UTR) and CAE2 encompassing the remainder of the nonstructural genes and 3′ UTR (NS2-3′ UTR). Deletions within CAE1 or CAE2 were not detected upon passage. We verified these results by two serial passages in C6/36 cells (see Fig. S8).

10.1128/mBio.01724-20.8 FIG S8 Passage of ZIKV RanDeL in C6/36 insect cells. Deletion depth profiles of passage 1 and 2 resemble landscapes from Vero cells. Download FIG S8, TIF file, 2.8 MB. Copyright © 2021 Notton et al. 2021 Notton et al. This content is distributed under the terms of the Creative Commons Attribution 4.0 International license.

## DISCUSSION

We describe a high-throughput method (RanDeL-seq) to comprehensively map viral *cis* and *trans* elements at a single-nucleotide resolution. RanDeL-seq takes advantage of *in vitro* transposition, dual exonuclease chewback, and barcode cassettes to make randomly distributed deletions of various size throughout a sequence of interest. As a proof of concept, we built and screened RanDeLs of >23,000 HIV-1 variants and >90,000 ZIKV variants. Tracking and sequencing barcodes at each stage of the scheme (transfection and passage to passage) revealed elements critical for different stages in the viral life cycle, particularly transmission, and enabled mapping of these elements in HIV-1 and ZIKV at single-base resolution.

The deletion landscape for HIV-1 recapitulated known CAEs (LTR, Ψ, and RRE) and their roles in transcription, encapsidation, and egress. Further analysis with traditional reverse genetics approaches validated that deletions in mapped *trans*-acting regions were able to be *trans*-complemented by HIV-1. Despite previous claims that the genomic RNA packaging enhancer (GRPE) is important for encapsidation (40), deletions of this region (nucleotide position 2022 to 2188) did not affect mobilization, in agreement with the findings of Nikolaitchik and Hu (75). The results also showed that the cPPT, despite its debated role in HIV-1 replication, was as important for sustained viral replication as the LTR, Ψ, and RRE. Surprisingly, we also identified a necessary 671-nucleotide region from the RRE to SA-7 which has not previously been reported and the function of which is undetermined. Previous work suggested that deletion to upstream cryptic splice sites 7a and 7b, but not SA-7, still allowed for HIV-1 replication (71). There were no instances of mutants where SA-7 remained intact, and SA-7a and -7b were deleted, as evidenced by the final HIV-1 deletion landscape. Additionally, SA-7 is heavily regulated, with an intronic splicing silencer (ISS), exonic splicing silencer (ESS), and exonic splicing enhancer (ESE). Interestingly, previous work suggested these elements were *cis*-acting (31, 76–78), but RanDeL-seq shows only the ISS is included in CAE3; therefore, deletion of the ESS and ESE was tolerated or could be provided in *trans*.

Analysis of ZIKV deletion landscape, in two different cell types, showed that deletions in the C protein, nonstructural genes NS2 to NS5, and UTRs are not tolerated and could not be supplemented in *trans* by the wild-type virus. This ZIKV profile adds to established flavivirus CAE and TAE models that focus on conserved elements of 5′ and 3′ UTRs (79), as seen with yellow fever virus (41), West Nile virus (80, 81), dengue virus (82, 83), and hepatitis C virus (84). While it accurately identifies the highly structured UTRs as critical CAEs, RanDeL-seq also demonstrated that deletions in C, NS5, and the other nonstructural proteins (NS2 to NS4) could not be complemented in *trans*. A recent study of the same strain of ZIKV reached similar conclusions through transposon insertion instead of deletion (85). Insertions in NS2 to NS5 were not tolerated, except at the regions proximal to the protein cleavage sites. However, that group found that insertions were tolerated in the C protein, which RanDeL-seq labeled as a CAE.

We note a number of limitations to RanDeL-seq. First, the one-pot method can create extremely diverse libraries *in vitro*, but transformation of E. coli limits the library diversity, due to selection against potentially “toxic” or unstable sequences in viral genomes. This limitation is shown by the finding that the initial deletion depths are not flat across the viral genome. Specific regions (i.e., *gag*/*pol* HIV deletions) are still selected for despite having less coverage in the initial construction and transfection. RanDeL-seq may also be too inefficient to produce diverse libraries for viruses with much larger genomes, such as herpesviruses (encoded on 250-kb bacterial artificial chromosomes [BACs]) (86). Transformation of bacteria with high-molecular-weight DNA is inefficient, and large genomes are easily damaged by shearing during the physical manipulations required for cleanup. However, libraries could be developed by dividing large genomes into smaller pieces that can be mutated separately and then reassembled using suitable methods such as REXER (87), and could be incorporated into other elegant frameworks for mapping DIPs (88).

The HIV-1 screen was conducted using a single molecular clone of HIV-1 and a single clonal cell line (MT-4). It is possible that CAEs vary between viral strains and between cell lines and tissue types. Conducting the screen in tissue explants (peripheral blood mononuclear cells [PBMCs] or human lymphoid aggregate cultures [HLACs]) may reveal different results. Also, the method is unable to monitor recombination between viruses (89), which could produce viral strains that have acquired more than one deletion and create linkage effects. Similarly, no sequencing outside the barcode cassette was conducted during serial passage, precluding the detection of additional mutations. However, we show a strong correlation between replicates, indicating that the observed selection was deterministic rather than a result of drift.

Compared to preexisting methods of CAE mapping, RanDeL-seq is able to cover the full viral genome with random deletions of variable size, track barcode (i.e., specific mutation) prevalence over time, and map at a single-nucleotide resolution. It is an improvement on methods of creating viral deletion mutants that rely on site-directed mutagenesis, iterative deletion, or spontaneous DI RNA emergence in culture; RanDeL-seq can comprehensively map full-length viruses, not just one targeted location. Additionally, RanDeL-seq fully abrogates genomic regions rather than silencing potential CAEs with single nucleotide polymorphisms (SNPs), stop codons, or sequence changes that do not affect protein synthesis. This full deletion allows determination of the essential nature of each genomic region.

The advantages of the method, along with its speed and low cost, make it attractive for studying novel, emerging viruses. The method can be rapidly deployed to identify CAEs for antiviral drug targeting, minimal sequences necessary for vaccine development, and candidates for novel antiviral therapies such as TIPs. Collectively, RanDeL-seq could be a valuable and versatile framework of general use to virology, aiding the study of viral replication mechanisms and the development of novel antiviral therapeutics.

## MATERIALS AND METHODS

### Plasmids.

pNL4-3 is a molecular clone of HIV-1 subtype B (90) and was a kind gift of Malcom Martin (AIDS reagent program, number 114). Two molecular clones of ZIKV, strain MR-766, were a generous gift from Matthew Evans. Two versions were available: a wild-type clone, pMR766(+), and a mutant, pMR766(−). The mutant clone has a GDD→GNN mutation in NS5 and lacks a functional RNA-dependent RNA polymerase.

### Cells.

All cells were grown at 37°C with 5% CO_2_. HEK 293T cells and C6/36 Aedes albopictus cells (American Type Culture Collection, numbers CRL-3216 and CRL-1660, respectively) were propagated in Dulbecco’s modified Eagle medium (DMEM) supplemented with 10% fetal bovine serum (FBS; Fisher Scientific) and 1% penicillin (pen)-streptomycin (strep) (Fisher Scientific), referred to as D10. Vero cells (African green monkey [Cercopithecus aethiops] kidney cells) (ATCC, number CCL-81) were also propagated in D10. MT-4 cells (NIH AIDS reagent program, number 120) were propagated in RPMI 1640 medium supplemented with 10% FBS, 1% pen-strep, HEPES, and l-glutamine, referred to as R10.

### Reagent sourcing.

All enzymes were obtained from New England Biolabs (NEB; Billerica, MA, USA) unless indicated otherwise. All chemicals were obtained from Sigma-Aldrich (St. Louis, MO, USA), unless indicated otherwise. DNA oligonucleotides and synthetic dsDNA were obtained from Integrated DNA Technologies (Coralville, IA, USA).

### Transposon DNA cassettes.

Transposon cassettes were ordered in 3 pieces as synthetic dsDNA (<500 bp) (gBlocks; IDT) and cloned by Gibson assembly into pUC19 (linearized at the BamHI site) (91). Postassembly, linear transposon cassettes were constructed by standard Q5 Hotstart PCR protocol (NEB) with TN5MK plasmid template and the oligonucleotides oTN5-F and oTN5-R (IDT). The template was amplified under the following conditions: 98°C for 30 s, 15 cycles of 98°C for 10 s, 68°C for 20 s, and 72°C for 50 s, final extension at 72°C for 5 min, and hold at 10°C. PCR products were purified on a column with a Zymo DCC-5 kit (Zymo Research) and then analyzed on a 0.8% agarose-Tris-EDTA (TE) gel. The 1.4-kb transposon cassettes were excised and cleaned using Qiagen gel extraction kit (Qiagen) and Zymo DNA columns.

### Barcode DNA cassettes.

HIV-1 barcodes were blunt-end, 5′-phosphorylated 60-bp DNA cassettes prepared by standard Q5 Hotstart PCR with BC20v1-F and BC20v1-R of the oligonucleotide pool BC20-T (IDT). Oligonucleotide sequences are provided in Table S2 in the supplemental material. BC20-T oligonucleotides were 60-bp ssDNA molecules with consensus 20-bp flanking sequences and a middle 20 bp with machine-mixed bases (random sequences for barcodes). Reactions were cycled at 98°C for 30 s, followed by 5 cycles of 98°C for 10 s and 65°C for 75 s, 1 cycle of 65°C for 5 min, and a hold at 10°C. Post-PCR, barcode cassettes were column purified (Zymo). A 3′ dT overhang was added with a 3′→5′ exonuclease-deficient Klenow fragment of E. coli DNA polymerase I per the manufacturer’s protocol. The reaction mixture was incubated at 37°C for 3 h. Postincubation, DNA was cleaned by column purification (Zymo) and eluted in Tris-acetate-EDTA buffer.

10.1128/mBio.01724-20.10 TABLE S2 Oligonucleotide sequences, listed from 5′ to 3′. Download Table S2, DOCX file, 0.1 MB. Copyright © 2021 Notton et al. 2021 Notton et al. This content is distributed under the terms of the Creative Commons Attribution 4.0 International license.

ZIKV libraries were prepared identically with a slight difference in the forward and reverse common sequences of the barcode cassette (BC20v2-F, BC20v2-R). These sequences modified a triple repeat in the forward barcode read (GGG) to avoid problems with the sequencing of low-diversity libraries on the Illumina HiSeq 4000.

### Chewback conditions.

Template DNA, λ-HindIII, was initially heated to 60°C for 3 min and immediately cooled to separate annealed cohesive cos ends. A standard 50-μl chewback reaction mixture was prepared on ice by combining distilled water (dH_2_O), 10× NEB2.1, λ-HindIII DNA template (500 ng/μl), T4 DNA polymerase (3 U/μl), RecJ_f_ (30 U/μl), and extreme thermostable (ET) SSB (500 ng/μl). The reaction mixture was then incubated at 37°C. After 30 min, 1 μl of 10 mM deoxynucleoside triphosphates (dNTPs) was added, and the reaction mixture was mixed and returned to 37°C for 11 min to allow T4 DNA polymerase to fill in recessed ends. The reaction was halted by adding EDTA (pH 8.0) to a final concentration of 20 mM. Various dropout reactions were conducted, where dH_2_O was substituted for enzymes.

### Determination of chewback rate.

A 4.3-kb dsDNA template was obtained by purifying the 4,361-bp fragment of the λ-HindIII digest. The λ-HindIII template was run on a 0.8% agarose gel, stained with SYBR Safe, and excised. DNA was recovered by adding 0.1 gel volumes of β-agarase I reaction buffer (NEB), melting gel slices briefly at 65°C, cooling to 42°C, and immediately adding 1 U of β-agarase I per 100 μl of molten gel (NEB). The mixture was incubated at 42°C for 60 min to release DNA bound in the agarose matrix. DNA was precipitated from the digested fraction with sodium acetate (3 M) and 2-propanol. After mixing, the reaction mixture was spun at 20,000 × *g* for 15 min at 25°C, and the supernatant was aspirated. The DNA pellet was washed once with 70% ethanol, allowed to air dry briefly, then dissolved in TE buffer.

A chewback reaction was set up per minimal conditions and incubated at 37°C. At 0, 5, 10, 15, 20, 25, 30, 40, 50, 60, 70, and 80 min of incubation, an aliquot of the reaction mixture was removed and combined with equal volumes dNTP buffer (NEB2.1, 10 mM dNTP, dH_2_O). These 12 reaction mixtures were then incubated at 37°C for 11 min to allow T4 DNA polymerase to fill in the single-stranded tails that remain uncleaved by RecJ_f_. After 11 min of fill-in, the reaction was halted with an equal volume of Stop buffer (EDTA, dH_2_O).

The concentration of dsDNA was determined by a fluorimetric method (PicoGreen; Thermo Fisher Scientific). Each reaction mixture was diluted in TE buffer and mixed with a PicoGreen working stock (diluted to 1/200 in TE buffer) to be read with an EnSpire plate reader (Perkin Elmer) with 480-nm excitation and a 520-nm emission filter. Fluorescence was compared to that of a λ DNA standard. All reactions were performed in triplicates. Chewback rates at 37°C were calculated by fitting the decay in dsDNA (fluorescence signal) at various time points to a linear regression model with the freely available R statistical software.

### Construction of RanDeL.

**(i) DNA extraction, precipitation, and wash.** Throughout construction of the random deletion libraries, DNA was extracted, precipitated, and washed with the same methods. DNA samples were extracted with 25:24:1 phenol-chloroform-isoamyl alcohol equilibrated with TE buffer, followed by a second extraction with pure chloroform (Sigma-Aldrich). The upper aqueous layer was transferred to a new DNA LoBind tube, and 25 μg of coprecipitating GenElute linear polyacrylamide (Sigma-Aldrich) was added and the solution mixed to homogeneity. DNA samples were precipitated from the aqueous phase by MgCl_2_-polyethylene glycol 8000 (PEG 8000) precipitation. Samples were adjusted to a final concentration of 12.5% (wt/vol) PEG 8000 and 20 mM MgCl_2_ by adding MgCl_2_ (1 M) and 50% (wt/vol) PEG 8000. Reaction mixtures were inverted and flicked to mix and then spun at 20,000 × *g* for 60 min in a refrigerated microcentrifuge (Eppendorf) at 25°C to pellet all precipitated DNA. After centrifugation, supernatants were removed and discarded. Freshly prepared 70% ethanol was added, and the reaction mixtures were mixed by inversion. Samples were spun at 20,000 × *g* for 2 min to collect the pellet, and the supernatant was aspirated and discarded. Additional ethanol was added to wash the pellet, and samples were spun again at 20,000 × *g* for 2 min to collect DNA pellets. All supernatants were carefully removed, and the pellets were dried briefly at room temperature (5 min) until no visible liquid remained. DNA samples were solubilized by adding TE buffer, incubating the tube at 42°C for 20 min, and mixed by flicking the tube.

**(ii) *In vitro* transposition.** Transposon cassettes were inserted into pNL4-3 by *in vitro* transposition with *EZ*-Tn*5* transposase (Epicentre) per the manufacturer’s protocol and with equal moles of plasmid template and transposon. After a 2-h incubation in reaction buffer at 37°C, the reaction was halted with 1% SDS solution and heated to 70°C for 10 min. The entire volume of the reaction mixture was transferred onto a 0.025-μm membrane floating on TE buffer. Drop dialysis was allowed to proceed for 1 h. Plasmids were electroporated into bacterial cells and selected with the encoded antibiotic resistances (carbenicillin and kanamycin). Plasmid DNA was obtained by Qiagen Maxiprep according to the manufacturer’s protocol.

**(iii) Transposon excision.** Inserted transposons were excised by treatment with either meganuclease I-SceI or I-CeuI in CutSmart buffer (NEB). Reaction mixtures were incubated at 37°C for 8 h, with brief mixing by inversion performed every 2 h. DNA was extracted with phenol-chloroform, precipitated by MgCl_2_-PEG 8000, and ethanol washed for the next stages.

**(iv) Chewback.** Substrate DNA was heated to 60°C for 3 min and immediately placed on ice to separate DNA aggregates in preparation of chewback. Four standard chewback reactions were prepared, each with a different chewback length (5, 10, 15, and 20 min). At the appropriate time, the designated reaction mixture was removed from 37°C incubation, and dNTPs were added. The reaction mixture was mixed and returned to 37°C to allow T4 DNA polymerase to fill in recessed ends. After 11 min of fill-in, the reaction was halted and placed on ice. All chewback reactions were pooled and then extracted with two phenol-chloroform extractions. The DNA was desalted by running through separate Sephacryl gel filtration columns (Microspin S-400 HR columns; GE Lifesciences).

**(v) End repair.** DNA was pooled and blunt ended by NEBNext end repair reaction module (NEB) with incubation at 20°C for 30 min. DNA was extracted with phenol-chloroform, precipitated with MgCl_2_-PEG 8000, and ethanol washed for the next stages.

**(vi) Addition of 3′ dA overhang to backbone.** A 3′ dA overhang was added to the purified blunt-end truncated linear pNL4-3 DNA with a 3′→5′ exonuclease-deficient Klenow fragment of E. coli DNA polymerase I (NEB). The reaction mixture was incubated at 37°C for 1 h and then heat inactivated (70°C for 20 min). Treatment with Antarctic phosphatase (NEB) per the manufacturer’s protocol dephosphorylated the 5′ ends (1 h at 37°C, 5 min at 70°C to deactivate). DNA was migrated on a 0.8% agarose gel and stained with SYBR Safe. All DNA vectors greater than 8 kb were excised, recovered with β-agarase, precipitated with sodium acetate and 2-propanol, and ethanol washed.

**(vii) Ligation of barcode cassettes and chewed vector.** 3′ dT-tailed barcode cassettes were ligated into a 3′ dA-tailed vector, and the DNA was circularized using T4 DNA Ligase in a PEG 6000-containing buffer (Quick ligation buffer; NEB). Ligation was performed at a 30:1 insert-vector molar ratio at bench temperature (24°C) for 2 h, and then the reaction was halted by adding EDTA (pH 8.0) and mixing. Next, proteinase K (800 U/ml) (NEB) was added, and the reaction mixture was mixed and incubated for 30 min at 37°C to cleave bound T4 DNA ligase from the DNA.

**(viii) Sealing of nicks in hemiligated DNA.**Nicked DNA was sealed by sequential treatment with T4 polynucleotide kinase (T4 PNK) and *Taq* DNA ligase. Hemiligated DNA was 5′ phosphorylated with T4 PNK in T4 DNA ligase reaction buffer (NEB) at 25°C for 30 min. Reaction mixtures were purified with AMPure XP beads (Beckman Coulter Genomics) and eluted with T4 DNA ligase master mix. The eluate was incubated at 37°C for 60 min to phosphorylate DNA at the nicked sites. The nicks were then sealed by treatment with *Taq* DNA ligase (NEB) in *Taq* DNA ligase reaction buffer at 75°C for 15 min. Ligated DNA was purified by AMPure XP beads and eluted in TE buffer.

**(ix) Library transformation and outgrowth.** The purified ligation was electroporated into electrocompetent E. coli (DH10B) cells. Cells were allowed to recover in SOC (Thermo Fisher) and then expanded for overnight growth in LB-Miller supplemented with carbenicillin. Finally, deletion library plasmid DNA was isolated from spun-down harvested cultures with a Qiagen Maxiprep.

### Transfection of viral stocks.

Cotransfections were with equal ratios of wild-type and deletion library plasmids. 293T cells were added to flasks at a ratio of 5e6 cells/ml in DMEM supplemented with 25 mM HEPES. Wild-type and deletion library plasmids were diluted in unsupplemented DMEM (i.e., no serum or antibiotics added) to a concentration of 10 ng/ml total DNA, and polyethyleneimine (PEI) was added to a concentration of 30 μg/ml in a volume ∼10% of the total volume in the transfection well or dish (e.g., 200 μl for a 6-well plate with 2 ml medium). The transfection mix was vortexed, incubated at bench temperature (24°C) for 15 min, and then added to the 293T flasks. Medium was replaced after an overnight incubation (16 to 20 h). Virus was harvested at either 48 h (HIV) or 72 h (ZIKV) posttransfection by passing through 0.45-μm sterile filters (Millipore). HIV-1 stocks were prepared with pNL4-3 and the pNL4-3 deletion library. ZIKV viral stocks were prepared with pMR766(+) and one of the four MR-766 deletion libraries: pMR766(+)ΔS, pMR766(+)ΔL, pMR766(−)ΔS, or pMR766(−)ΔL.

### HIV high-MOI screen.

**(i) Concentration of virus.** Concentrated virus was prepared by ultracentrifugation (Beckman Coulter Optima XE-90, rotor SW 28) at 20,000 rpm through a 6% iodixanol gradient (Sigma-Aldrich, D1556, 250 ml) for 1.5 to 2 h at 4°C.

**(ii) Titration of viral stocks.** The concentrated HIV-1 stocks were titrated by infecting cultures of MT-4 with concentrated virus and scoring for HIV p24-producing cells at 24 h postinfection. Virus was added to MT-4 cells in R10, mixed briefly, and then incubated for 4 h at 37°C. After 4 h, additional medium was added, and the infection was allowed to proceed for an additional 20 h (a single round of replication). At 24 h postinfection, cultures were fixed with 20% formaldehyde (tousimis) and incubated for at least 1 h at 4°C. After fixing, cells were permeabilized by treatment with 75% ice-cold methanol for 10 min and then stained with a phycoerythrin-labeled monoclonal antibody (KC57-RD1; BD) for 30 min before washing once in stain buffer. At least 50,000 live cells were counted by flow cytometry on a FACSCalibur DxP8. Gates were drawn based upon a stained naive cell population. Analysis was conducted in FlowJo.

**(iii) High-MOI passage scheme.** On day 0, 2 × 10^6^ MT-4 cells were infected at an MOI of 5 to 20 with the prepared and titrated virus pool for 4 h in a volume of 2 ml and then transferred to a T25 flask containing 10 ml of MT-4 cells at a concentration of 10^6^ cells/ml. On day 2 (40 h postinfection [hpi]), the 12 ml of culture was transferred to a T175 flask containing 60 ml of MT-4 cells in R10 at a concentration of 10^6^ cells/ml. On day 3 (70 to 72 hpi), supernatant from the MT-4 was clarified by centrifugation and 0.45-μm filtration and then concentrated by ultracentrifugation as described above. One cycle corresponds to 3 rounds of HIV-1 replication (completed on day 1, day 2, and day 3) and was repeated four times for a total of 12 passages (i.e., rounds of replication). The cycle was repeated a total of four times (12 passages/rounds of replication) with 3 biological replicates (K, L, and M). Wild-type pNL4-3 controls were passaged alongside the deletion library, also in triplicates (A, B, and C).

**(iv) Viral RNA isolation.** Viral RNA was isolated from the concentrated virus pool at passage 0, passage 3, passage 6, passage 9, and passage 12 using a QIAamp viral RNA minikit (Qiagen) per the manufacturer’s instructions with two exceptions: (i) carrier RNA was replaced with 5 of linear polyacrylamide (Sigma) per isolation, and (ii) 5⋅× 10^6^ copies of bacteriophage MS2 RNA (Roche) were spiked in per isolation. Total cellular RNA from 293T cells was isolated using TRIzol (Life Technologies) from cell pellets obtained at the time of viral harvest. A poly(A) fraction, representing mRNA, was isolated by annealing total RNA to magnetic d(T)_25_ beads to pull down polyadenylated transcripts [NEBNext poly(A) mRNA magnetic isolation module].

**(v) RT-qPCR analysis.** Purified vRNA was reverse-transcribed with Superscript III (Thermo Fisher) and random primer mix (New England Biolabs) for quantification by RT-qPCR with Fast SYBR green master mix (Thermo Fisher). Barcode cassettes were quantified by oligonucleotides BC20v1-F and BC20v1-R. Total HIV RNA was estimated by primers targeting HIV *pol*, NL43pol-F and NL43pol-R. Samples were normalized for recovery by determining levels of MS2 RNA recovered by oligonucleotides MS2-F and MS2-R (sequences from reference 92). Relative expression was calculated by traditional RT-qPCR methods (93). Oligonucleotide sequences can be found in Table S2.

### ZIKV high-MOI screen.

**(i) Concentration of viral stocks.** Virus stocks were concentrated by ultrafiltration. Clarified supernatant was added to a 100-kDa molecular-weight-cutoff (MWCO) filtration device in 20 aliquots. The device was spun at 1,200 × *g* for 20 to 30 min until the concentrate volume was less than 1 ml. The flowthrough fraction was removed, an additional supernatant was added to the upper reservoir, and the process was repeated. Generally, clarified supernatant was concentrated 20× to 40×. Concentrated stocks were adjusted to 20% (vol/vol) FBS and 10 mM HEPES (to reduce loss in infectivity from freeze-thawing).

**(ii) Titration of viral stocks.** ZIKV stocks were titrated by plaque assay (94). On the day before infection, Vero cells were seeded in 6-well or 12-plates and cultured to approximately 50% confluence. On the day of infection (0 dpi), serial 10-fold dilutions of sample stocks were prepared by dilution in DMEM supplemented with 3% (vol/vol) heat-inactivated FBS. The medium from each well of the infection plate was removed and replaced with serially diluted virus. The plate was gently rocked and returned to the incubator for a period of 1 h, with gently rocking applied every 15 min. After 1 h of adsorption, the virus was removed and the cultures overlaid with a viscous solution of 1% (wt/vol) carboxymethylcellulose (Sigma number C4888) in DMEM-F12 (8% FBS, 1% pen-strep). Infection plates were returned to the incubator and left undisturbed for 5 days. At 5 dpi, the wells were with 20% formaldehyde and mixed gently for 1 h. The supernatant was removed, and the culture was stained with a solution of 1% crystal violet in 20% ethanol for 15 min. Wells were destained by rinsing with dH_2_O. Plaques were 1 to 2 mm in diameter and could be visualized as clear circular patches on the stained purple monolayer.

**(iii) High-MOI passage scheme.** On day 0, Vero cells were infected at an MOI of 16 to 30 with a virus pool containing wild-type ZIKV and ZIKV deletion libraries. The inoculum was applied in a low volume in a 6-well plate for 1 h and then removed. Supernatant was collected at 1, 2, and 3 dpi, corresponding to one passage. Virus from passage 1 was titrated by plaque assay and used to infect Vero cells for passage 2. The passage scheme was conducted with 2 biological replicates.

**(iv) Viral RNA isolation.** ZIKV Viral RNA was isolated from the concentrated virus pool at 293T transfection, passage 1, and passage 2 per similar methods to those for the HIV screen.

**(v) RT-qPCR analysis.** Purified RNA was reverse-transcribed with MuLV-R (NEB) and random primer mix (NEB) for quantification by RT-qPCR with SYBR green master mix. Barcode cassettes were quantified by oligonucleotides BC20v2-F and BC20v2-R. Total ZIKV RNA was estimated by primers targeting the ZIKV capsid protein (ZIK-C), MR766-C-F and MR766-C-R. Samples were normalized for recovery by determining levels of MS2 RNA recovered by oligonucleotides MS2-F and MS2-R. Relative expression was calculated as conducted in the HIV-1 screen. Oligonucleotide sequences can be found in Table S2.

### Next-generation sequencing analysis.

**(i) Genotyping of plasmid libraries.** Insertion and deletion plasmid libraries were prepared for paired-end sequencing on the Illumina HiSeq/MiSeq platforms by a Nextera XT kit (Illumina) from 1 ng of each library. Transposon insertion and PCR enrichment were performed per the manufacturer’s instructions, but the sublibraries were pooled and size selected by running on a 1.5% agarose gel, staining with SYBR Safe (Thermo Fisher), and excising a gel fragment corresponding to DNA of a size range of 350 to 500 bp. DNA was purified from the gel slice using Qiagen buffer QG, buffer PE (Qiagen), and DCC-5 columns (Zymo Research). The sublibraries were pooled and sequenced on a single lane of a HiSeq 4000 (Illumina), using 2 × 125-bp reads.

Transposon insertion locations were computed by filtering for high-quality reads containing an exact match of either mosaic end sequence of TN5MK and then extracting flanking the regions to build an insertion map. A lookup table matching deletion locus to barcode sequence was determined by (i) searching reads for the forward and reverse common barcode sequences and extracting the intermediate 20 bp, (ii) assembling a list of barcode sequences, and (iii) assigning flanking regions to each barcoded deletion using custom Python software.

**(ii) Sequencing of serial passage.** Illumina sequencing libraries were prepared by a modification of a method specified in reference 95 and detailed in Fig. S5. Barcode cassettes were amplified using a minimum number of cycles (typically 12 to 18) to prevent overamplification (post-log-phase PCR). Illumina adaptors were added by two rounds of PCR (5 cycles each) to add phasing adaptors, random barcodes, and multiplexing barcodes. Sublibraries were size selected on 5% Tris-borate-EDTA (TBE) polyacrylamide gels and pooled for sequencing. Twenty to 30 sublibraries were sequenced on two lanes of a HiSeq 4000 (Illumina) (spiked with 25% PhiX), using a single 1 × 50-bp read at the Center for Advanced Technology at University of California, San Francisco. Barcodes were tallied using custom Python software and matched to deletion loci using the lookup table prepared previously to calculate deletion depth.

### Mobilization of deletion vector assays.

Deletion vectors were packaged by transfection of 293T cells with common lentiviral viral vectors delta8.9 and VSV-G. Supernatant was harvested at 48 h posttransfection, filtered with 0.45-μm sterile filters, concentrated via ultracentrifugation, and used to transduce MT-4 cells. Four days postransduction, cells were split and superinfected by HIV-1 wild-type virus or a medium negative control. Two days postsuperinfection, supernatant was filtered and transferred onto naive MT-4 cells. After 2 more days of growth, supernatant viral RNA was isolated and quantified by RT-qPCR per previous methods and primers (BC, MS2).

Single-copy cell lines of each cloned deletion vector were grown out and superinfected with HIV-1 wild-type virus or medium negative control. Three days postsuperinfection, supernatant was collected, filtered, and transferred onto naive MT-4 cells. After 2 more days of growth, genomic DNA was isolated using a commercial kit (NucleoSpin Blood). PCR to detect A, B, and C blocks (primers in Table S2) was performed with One*Taq* DNA polymerase under the following conditions: 95°C for 5 min s, 35 cycles of 94°C for 15 s, 61°C for 30 s, and 68°C for 90 s, a final extension at 68°C for 5 min, and hold at 10°C. PCR products were purified on a column with Zymo DCC-5 kit (Zymo Research) and then analyzed on a 0.8% agarose-TE gel.

### Data availability.

The data that support the findings of this study are available from the corresponding author upon reasonable request. All unique biological materials are available from the corresponding author. Custom code is available upon request.

## References

[1] Jacob F, Monod J 1961 Genetic regulatory mechanisms in the synthesis of proteins. J Mol Biol 3:318–356. doi:10.1016/s0022-2836(61)80072-7. 13718526

[2] Nicholson BL, White KA 2014 Functional long-range RNA-RNA interactions in positive-strand RNA viruses. Nat Rev Microbiol 12:493–504. doi:10.1038/nrmicro3288. 24931042 PMC7097572

[3] Newburn LR, White KA 2015 *cis*-acting RNA elements in positive-strand RNA plant virus genomes. Virology 479-480:434–443. doi:10.1016/j.virol.2015.02.032. 25759098

[4] Romero-Lopez C, Berzal-Herranz A 2013 Unmasking the information encoded as structural motifs of viral RNA genomes: a potential antiviral target. Rev Med Virol 23:340–354. doi:10.1002/rmv.1756. 23983005 PMC7169113

[5] Liu Y, Wimmer E, Paul AV 2009 *cis*-acting RNA elements in human and animal plus-strand RNA viruses. Biochim Biophys Acta 1789:495–517. doi:10.1016/j.bbagrm.2009.09.007. 19781674 PMC2783963

[6] Lim CS, Brown CM 2017 Know your enemy: successful bioinformatic approaches to predict functional RNA structures in viral RNAs. Front Microbiol 8:2582. doi:10.3389/fmicb.2017.02582. 29354101 PMC5758548

[7] Chen IH, Huang YW, Tsai CH 2017 The functional roles of the *cis*-acting elements in bamboo mosaic virus RNA genome. Front Microbiol 8:645. doi:10.3389/fmicb.2017.00645. 28450857 PMC5390519

[8] Hermann T 2016 Small molecules targeting viral RNA. Wiley Interdiscip Rev RNA 7:726–743. doi:10.1002/wrna.1373. 27307213 PMC7169885

[9] Gasparian AV, Neznanov N, Jha S, Galkin O, Moran JJ, Gudkov AV, Gurova KV, Komar AA 2010 Inhibition of encephalomyocarditis virus and poliovirus replication by quinacrine: implications for the design and discovery of novel antiviral drugs. J Virol 84:9390–9397. doi:10.1128/JVI.02569-09. 20631142 PMC2937643

[10] Iwasaki M, Ngo N, Cubitt B, Teijaro JR, de la Torre JC 2015 General molecular strategy for development of arenavirus live-attenuated vaccines. J Virol 89:12166–12177. doi:10.1128/JVI.02075-15. 26401045 PMC4645318

[11] Pena L, Sutton T, Chockalingam A, Kumar S, Angel M, Shao H, Chen H, Li W, Perez DR 2013 Influenza viruses with rearranged genomes as live-attenuated vaccines. J Virol 87:5118–5127. doi:10.1128/JVI.02490-12. 23449800 PMC3624320

[12] Jang YH, Seong BL 2012 Principles underlying rational design of live attenuated influenza vaccines. Clin Exp Vaccine Res 1:35–49. doi:10.7774/cevr.2012.1.1.35. 23596576 PMC3623510

[13] Graham RL, Deming DJ, Deming ME, Yount BL, Baric RS 2018 Evaluation of a recombination-resistant coronavirus as a broadly applicable, rapidly implementable vaccine platform. Commun Biol 1:179. doi:10.1038/s42003-018-0175-7. 30393776 PMC6206136

[14] Perez JT, Pham AM, Lorini MH, Chua MA, Steel J, tenOever BR 2009 MicroRNA-mediated species-specific attenuation of influenza A virus. Nat Biotechnol 27:572–576. doi:10.1038/nbt.1542. 19483680

[15] Tanner EJ, Kirkegaard KA, Weinberger LS 2016 Exploiting genetic interference for antiviral therapy. PLoS Genet 12:e1005986. doi:10.1371/journal.pgen.1005986. 27149616 PMC4858160

[16] Huang AS, Baltimore D 1970 Defective viral particles and viral disease processes. Nature 226:325–327. doi:10.1038/226325a0. 5439728

[17] Von Magnus P 1954 Incomplete forms of influenza virus. Adv Virus Res 2:59–79. doi:10.1016/s0065-3527(08)60529-1. 13228257

[18] Knipe DM, Howley PM 2013 Fields virology, 6th ed Lippincott Williams & Wilkins, Philadelphia, PA.

[19] Henle W, Henle G 1943 Interference of inactive virus with the propagation of virus of influenza. Science 98:87–89. doi:10.1126/science.98.2534.87. 17749157

[20] Frensing T, Pflugmacher A, Bachmann M, Peschel B, Reichl U 2014 Impact of defective interfering particles on virus replication and antiviral host response in cell culture-based influenza vaccine production. Appl Microbiol Biotechnol 98:8999–9008. doi:10.1007/s00253-014-5933-y. 25132064

[21] Poirier EZ, Mounce BC, Rozen-Gagnon K, Hooikaas PJ, Stapleford KA, Moratorio G, Vignuzzi M 2015 Low-fidelity polymerases of alphaviruses recombine at higher rates to overproduce defective interfering particles. J Virol 90:2446–2454. doi:10.1128/JVI.02921-15. 26676773 PMC4810721

[22] Rezelj VV, Levi LI, Vignuzzi M 2018 The defective component of viral populations. Curr Opin Virol 33:74–80. doi:10.1016/j.coviro.2018.07.014. 30099321

[23] Kupke SY, Riedel D, Frensing T, Zmora P, Reichl U 2018 A novel type of influenza A virus-derived defective interfering particle with nucleotide substitutions in its genome. J Virol 93:e01786-18. doi:10.1128/JVI.01786-18. PMC6364022 30463972

[24] Dimmock NJ, Easton AJ 2014 Defective interfering influenza virus RNAs: time to reevaluate their clinical potential as broad-spectrum antivirals? J Virol 88:5217–5227. doi:10.1128/JVI.03193-13. 24574404 PMC4019098

[25] Dimmock NJ, Rainsford EW, Scott PD, Marriott AC 2008 Influenza virus protecting RNA: an effective prophylactic and therapeutic antiviral. J Virol 82:8570–8578. doi:10.1128/JVI.00743-08. 18579602 PMC2519629

[26] Beigel JH, Nam HH, Adams PL, Krafft A, Ince WL, El-Kamary SS, Sims AC 2019 Advances in respiratory virus therapeutics - A meeting report from the 6th isirv Antiviral Group conference. Antiviral Res 167:45–67. doi:10.1016/j.antiviral.2019.04.006. 30974127 PMC7132446

[27] Wolter F, Puchta H 2018 Application of CRISPR/Cas to understand *cis*- and *trans*-regulatory elements in plants. Methods Mol Biol 1830:23–40. doi:10.1007/978-1-4939-8657-6_2. 30043362

[28] Morelli A, Cabezas Y, Mills LJ, Seelig B 2017 Extensive libraries of gene truncation variants generated by in vitro transposition. Nucleic Acids Res 45:e78. doi:10.1093/nar/gkx030. 28130425 PMC5449547

[29] Pocock GM, Zimdars LL, Yuan M, Eliceiri KW, Ahlquist P, Sherer NM 2017 Diverse activities of viral *cis*-acting RNA regulatory elements revealed using multicolor, long-term, single-cell imaging. Mol Biol Cell 28:476–487. doi:10.1091/mbc.E16-08-0612. 27903772 PMC5341730

[30] Diao Y, Fang R, Li B, Meng Z, Yu J, Qiu Y, Lin KC, Huang H, Liu T, Marina RJ, Jung I, Shen Y, Guan KL, Ren B 2017 A tiling-deletion-based genetic screen for cis-regulatory element identification in mammalian cells. Nat Methods 14:629–635. doi:10.1038/nmeth.4264. 28417999 PMC5490986

[31] Takata MA, Soll SJ, Emery A, Blanco-Melo D, Swanstrom R, Bieniasz PD 2018 Global synonymous mutagenesis identifies *cis*-acting RNA elements that regulate HIV-1 splicing and replication. PLoS Pathog 14:e1006824. doi:10.1371/journal.ppat.1006824. 29377940 PMC5805364

[32] Smyth RP, Despons L, Huili G, Bernacchi S, Hijnen M, Mak J, Jossinet F, Weixi L, Paillart JC, von Kleist M, Marquet R 2015 Mutational interference mapping experiment (MIME) for studying RNA structure and function. Nat Methods 12:866–872. doi:10.1038/nmeth.3490. 26237229

[33] Smyth RP, Smith MR, Jousset AC, Despons L, Laumond G, Decoville T, Cattenoz P, Moog C, Jossinet F, Mougel M, Paillart JC, von Kleist M, Marquet R 2018 In cell mutational interference mapping experiment (in cell MIME) identifies the 5' polyadenylation signal as a dual regulator of HIV-1 genomic RNA production and packaging. Nucleic Acids Res 46:e57. doi:10.1093/nar/gky152. 29514260 PMC5961354

[34] Firth AE 2014 Mapping overlapping functional elements embedded within the protein-coding regions of RNA viruses. Nucleic Acids Res 42:12425–12439. doi:10.1093/nar/gku981. 25326325 PMC4227794

[35] Chen MH, Frey TK 1999 Mutagenic analysis of the 3' *cis*-acting elements of the rubella virus genome. J Virol 73:3386–3403. doi:10.1128/JVI.73.4.3386-3403.1999. 10074193 PMC104103

[36] Goodfellow I, Chaudhry Y, Richardson A, Meredith J, Almond JW, Barclay W, Evans DJ 2000 Identification of a *cis*-acting replication element within the poliovirus coding region. J Virol 74:4590–4600. doi:10.1128/jvi.74.10.4590-4600.2000. 10775595 PMC111979

[37] Guo J, Han J, Lin J, Finer J, Dorrance A, Qu F 2017 Functionally interchangeable *cis*-acting RNA elements in both genome segments of a picorna-like plant virus. Sci Rep 7:1017. doi:10.1038/s41598-017-01243-z. 28432346 PMC5430698

[38] Liu ZY, Yu JY, Huang XY, Fan H, Li XF, Deng YQ, Ji X, Cheng ML, Ye Q, Zhao H, Han JF, An XP, Jiang T, Zhang B, Tong YG, Qin CF 2017 Characterization of *cis*-acting RNA elements of Zika virus by using a self-splicing ribozyme-dependent infectious clone. J Virol 91:e00484-14. doi:10.1128/JVI.00484-17. 28814522 PMC5640849

[39] Moomau C, Musalgaonkar S, Khan YA, Jones JE, Dinman JD 2016 Structural and functional characterization of programmed ribosomal frameshift signals in West Nile virus strains reveals high structural plasticity among *cis*-acting RNA elements. J Biol Chem 291:15788–15795. doi:10.1074/jbc.M116.735613. 27226636 PMC4957060

[40] Chamanian M, Purzycka KJ, Wille PT, Ha JS, McDonald D, Gao Y, Le Grice SF, Arts EJ 2013 A cis-acting element in retroviral genomic RNA links Gag-Pol ribosomal frameshifting to selective viral RNA encapsidation. Cell Host Microbe 13:181–192. doi:10.1016/j.chom.2013.01.007. 23414758 PMC3587049

[41] Corver J, Lenches E, Smith K, Robison RA, Sando T, Strauss EG, Strauss JH 2003 Fine mapping of a *cis*-acting sequence element in yellow fever virus RNA that is required for RNA replication and cyclization. J Virol 77:2265–2270. doi:10.1128/jvi.77.3.2265-2270.2003. 12525663 PMC140906

[42] Lin YJ, Liao CL, Lai MM 1994 Identification of the *cis*-acting signal for minus-strand RNA synthesis of a murine coronavirus: implications for the role of minus-strand RNA in RNA replication and transcription. J Virol 68:8131–8140. doi:10.1128/JVI.68.12.8131-8140.1994. 7966604 PMC237278

[43] Trobridge G, Josephson N, Vassilopoulos G, Mac J, Russell DW 2002 Improved foamy virus vectors with minimal viral sequences. Mol Ther 6:321–328. doi:10.1006/mthe.2002.0672. 12231167

[44] van Ooij MJ, Polacek C, Glaudemans DH, Kuijpers J, van Kuppeveld FJ, Andino R, Agol VI, Melchers WJ 2006 Polyadenylation of genomic RNA and initiation of antigenomic RNA in a positive-strand RNA virus are controlled by the same *cis*-element. Nucleic Acids Res 34:2953–2965. doi:10.1093/nar/gkl349. 16738134 PMC1474053

[45] Kirkegaard K, Nelsen B 1990 Conditional poliovirus mutants made by random deletion mutagenesis of infectious cDNA. J Virol 64:185–194. doi:10.1128/JVI.64.1.185-194.1990. 2152811 PMC249081

[46] Krishnakumar R, Grose C, Haft DH, Zaveri J, Alperovich N, Gibson DG, Merryman C, Glass JI 2014 Simultaneous non-contiguous deletions using large synthetic DNA and site-specific recombinases. Nucleic Acids Res 42:e111. doi:10.1093/nar/gku509. 24914053 PMC4132700

[47] Fulton BO, Sachs D, Beaty SM, Won ST, Lee B, Palese P, Heaton NS 2015 Mutational analysis of measles virus suggests constraints on antigenic variation of the glycoproteins. Cell Rep 11:1331–1338. doi:10.1016/j.celrep.2015.04.054. 26004185 PMC4464907

[48] Heaton NS, Sachs D, Chen CJ, Hai R, Palese P 2013 Genome-wide mutagenesis of influenza virus reveals unique plasticity of the hemagglutinin and NS1 proteins. Proc Natl Acad Sci U S A 110:20248–20253. doi:10.1073/pnas.1320524110. 24277853 PMC3864309

[49] Iverson EA, Goodman DA, Gorchels ME, Stedman KM 2017 Extreme mutation tolerance: nearly half of the archaeal fusellovirus Sulfolobus spindle-shaped virus 1 genes are not required for virus function, including the minor capsid protein gene *vp3*. J Virol 91:e02406-16. doi:10.1128/JVI.02406-16. 28148789 PMC5411595

[50] Wang L, Liu SY, Chen HW, Xu J, Chapon M, Zhang T, Zhou F, Wang YE, Quanquin N, Wang G, Tian X, He Z, Liu L, Yu W, Sanchez DJ, Liang Y, Jiang T, Modlin R, Bloom BR, Li Q, Deng JC, Zhou P, Qin FX, Cheng G 2017 Generation of a live attenuated influenza vaccine that elicits broad protection in mice and ferrets. Cell Host Microbe 21:334–343. doi:10.1016/j.chom.2017.02.007. 28279345

[51] Levy SF, Blundell JR, Venkataram S, Petrov DA, Fisher DS, Sherlock G 2015 Quantitative evolutionary dynamics using high-resolution lineage tracking. Nature 519:181–186. doi:10.1038/nature14279. 25731169 PMC4426284

[52] Bhasin A, Goryshin IY, Steiniger-White M, York D, Reznikoff WS 2000 Characterization of a Tn*5* pre-cleavage synaptic complex. J Mol Biol 302:49–63. doi:10.1006/jmbi.2000.4048. 10964560

[53] Goryshin IY, Reznikoff WS 1998 Tn*5 in vitro* transposition. J Biol Chem 273:7367–7374. doi:10.1074/jbc.273.13.7367. 9516433

[54] Reznikoff WS, Goryshin IY, Jendrisak JJ 2004 Tn*5* as a molecular genetics tool: *in vitro* transposition and the coupling of *in vitro* technologies with *in vivo* transposition. Methods Mol Biol 260:83–96. doi:10.1385/1-59259-755-6:083. 15020804

[55] McDonald R, Burnett V 2005 Novel single-round PCR and cloning of full-length envelope genes of HIV-1 may yield new insight into biomolecular antibacterial drug development. J Virol Methods 126:111–118. doi:10.1016/j.jviromet.2005.01.027. 15847926

[56] Muller HJ 1932 Some genetic aspects of sex. Am Nat 66:118–138. doi:10.1086/280418.

[57] Smith JM 1978 Optimization theory in evolution. Annu Rev Ecol Syst 9:31–56. doi:10.1146/annurev.es.09.110178.000335.

[58] Jayaraman B, Crosby DC, Homer C, Ribeiro I, Mavor D, Frankel AD 2014 RNA-directed remodeling of the HIV-1 protein Rev orchestrates assembly of the Rev-Rev response element complex. Elife 3:e04120. doi:10.7554/eLife.04120. 25486594 PMC4360532

[59] Hansen MMK, Wen WY, Ingerman E, Razooky BS, Thompson CE, Dar RD, Chin CW, Simpson ML, Weinberger LS 2018 A post-transcriptional feedback mechanism for noise suppression and fate stabilization. Cell 173:1609–1621 e15. doi:10.1016/j.cell.2018.04.005. 29754821 PMC6044448

[60] Van Maele B, De Rijck J, De Clercq E, Debyser Z 2003 Impact of the central polypurine tract on the kinetics of human immunodeficiency virus type 1 vector transduction. J Virol 77:4685–4694. doi:10.1128/jvi.77.8.4685-4694.2003. 12663775 PMC152151

[61] Riviere L, Darlix JL, Cimarelli A 2010 Analysis of the viral elements required in the nuclear import of HIV-1 DNA. J Virol 84:729–739. doi:10.1128/JVI.01952-09. 19889772 PMC2798343

[62] Zennou V, Petit C, Guetard D, Nerhbass U, Montagnier L, Charneau P 2000 HIV-1 genome nuclear import is mediated by a central DNA flap. Cell 101:173–185. doi:10.1016/S0092-8674(00)80828-4. 10786833

[63] Charneau P, Clavel F 1991 A single-stranded gap in human immunodeficiency virus unintegrated linear DNA defined by a central copy of the polypurine tract. J Virol 65:2415–2421. doi:10.1128/JVI.65.5.2415-2421.1991. 2016765 PMC240594

[64] Arhel N, Munier S, Souque P, Mollier K, Charneau P 2006 Nuclear import defect of human immunodeficiency virus type 1 DNA flap mutants is not dependent on the viral strain or target cell type. J Virol 80:10262–10269. doi:10.1128/JVI.00974-06. 17005705 PMC1617309

[65] Hu C, Saenz DT, Fadel HJ, Walker W, Peretz M, Poeschla EM 2010 The HIV-1 central polypurine tract functions as a second line of defense against APOBEC3G/F. J Virol 84:11981–11993. doi:10.1128/JVI.00723-10. 20844042 PMC2977901

[66] Yamashita M, Emerman M 2005 The cell cycle independence of HIV infections is not determined by known karyophilic viral elements. PLoS Pathog 1:e18. doi:10.1371/journal.ppat.0010018. 16292356 PMC1283251

[67] Dvorin JD, Bell P, Maul GG, Yamashita M, Emerman M, Malim MH 2002 Reassessment of the roles of integrase and the central DNA flap in human immunodeficiency virus type 1 nuclear import. J Virol 76:12087–12096. doi:10.1128/jvi.76.23.12087-12096.2002. 12414950 PMC136890

[68] Marsden MD, Zack JA 2007 Human immunodeficiency virus bearing a disrupted central DNA flap is pathogenic *in vivo*. J Virol 81:6146–6150. doi:10.1128/JVI.00203-07. 17392373 PMC1900283

[69] Ocwieja KE, Sherrill-Mix S, Mukherjee R, Custers-Allen R, David P, Brown M, Wang S, Link DR, Olson J, Travers K, Schadt E, Bushman FD 2012 Dynamic regulation of HIV-1 mRNA populations analyzed by single-molecule enrichment and long-read sequencing. Nucleic Acids Res 40:10345–10355. doi:10.1093/nar/gks753. 22923523 PMC3488221

[70] Sertznig H, Hillebrand F, Erkelenz S, Schaal H, Widera M 2018 Behind the scenes of HIV-1 replication: alternative splicing as the dependency factor on the quiet. Virology 516:176–188. doi:10.1016/j.virol.2018.01.011. 29407375

[71] Purcell DF, Martin MA 1993 Alternative splicing of human immunodeficiency virus type 1 mRNA modulates viral protein expression, replication, and infectivity. J Virol 67:6365–6378. doi:10.1128/JVI.67.11.6365-6378.1993. 8411338 PMC238071

[72] Schwarz MC, Sourisseau M, Espino MM, Gray ES, Chambers MT, Tortorella D, Evans MJ 2016 Rescue of the 1947 Zika virus prototype strain with a cytomegalovirus promoter-driven cDNA clone. mSphere 1:e00246-16. doi:10.1128/mSphere.00246-16. 27704051 PMC5040786

[73] Li D, Aaskov J, Lott WB 2011 Identification of a cryptic prokaryotic promoter within the cDNA encoding the 5' end of dengue virus RNA genome. PLoS One 6:e18197. doi:10.1371/journal.pone.0018197. 21483867 PMC3069047

[74] Jones CT, Patkar CG, Kuhn RJ 2005 Construction and applications of yellow fever virus replicons. Virology 331:247–259. doi:10.1016/j.virol.2004.10.034. 15629769

[75] Nikolaitchik OA, Hu WS 2014 Deciphering the role of the Gag-Pol ribosomal frameshift signal in HIV-1 RNA genome packaging. J Virol 88:4040–4046. doi:10.1128/JVI.03745-13. 24453371 PMC3993742

[76] Stoltzfus CM, Madsen JM 2006 Role of viral splicing elements and cellular RNA binding proteins in regulation of HIV-1 alternative RNA splicing. Curr HIV Res 4:43–55. doi:10.2174/157016206775197655. 16454710

[77] Grewe B, Ehrhardt K, Hoffmann B, Blissenbach M, Brandt S, Uberla K 2012 The HIV-1 Rev protein enhances encapsidation of unspliced and spliced, RRE-containing lentiviral vector RNA. PLoS One 7:e48688. doi:10.1371/journal.pone.0048688. 23133650 PMC3486793

[78] Tange TO, Damgaard CK, Guth S, Valcarcel J, Kjems J 2001 The hnRNP A1 protein regulates HIV-1 tat splicing via a novel intron silencer element. EMBO J 20:5748–5758. doi:10.1093/emboj/20.20.5748. 11598017 PMC125679

[79] Liu ZY, Li XF, Jiang T, Deng YQ, Zhao H, Wang HJ, Ye Q, Zhu SY, Qiu Y, Zhou X, Qin ED, Qin CF 2013 Novel *cis*-acting element within the capsid-coding region enhances flavivirus viral-RNA replication by regulating genome cyclization. J Virol 87:6804–6818. doi:10.1128/JVI.00243-13. 23576500 PMC3676100

[80] Zhang B, Dong H, Zhou Y, Shi PY 2008 Genetic interactions among the West Nile virus methyltransferase, the RNA-dependent RNA polymerase, and the 5' stem-loop of genomic RNA. J Virol 82:7047–7058. doi:10.1128/JVI.00654-08. 18448528 PMC2446981

[81] Lo MK, Tilgner M, Bernard KA, Shi PY 2003 Functional analysis of mosquito-borne flavivirus conserved sequence elements within 3' untranslated region of West Nile virus by use of a reporting replicon that differentiates between viral translation and RNA replication. J Virol 77:10004–10014. doi:10.1128/jvi.77.18.10004-10014.2003. 12941911 PMC224605

[82] Men R, Bray M, Clark D, Chanock RM, Lai CJ 1996 Dengue type 4 virus mutants containing deletions in the 3' noncoding region of the RNA genome: analysis of growth restriction in cell culture and altered viremia pattern and immunogenicity in rhesus monkeys. J Virol 70:3930–3937. doi:10.1128/JVI.70.6.3930-3937.1996. 8648730 PMC190271

[83] Manzano M, Reichert ED, Polo S, Falgout B, Kasprzak W, Shapiro BA, Padmanabhan R 2011 Identification of *cis*-acting elements in the 3'-untranslated region of the dengue virus type 2 RNA that modulate translation and replication. J Biol Chem 286:22521–22534. doi:10.1074/jbc.M111.234302. 21515677 PMC3121397

[84] Arumugaswami V, Remenyi R, Kanagavel V, Sue EY, Ngoc Ho T, Liu C, Fontanes V, Dasgupta A, Sun R 2008 High-resolution functional profiling of hepatitis C virus genome. PLoS Pathog 4:e1000182. doi:10.1371/journal.ppat.1000182. 18927624 PMC2564836

[85] Fulton BO, Sachs D, Schwarz MC, Palese P, Evans MJ 2017 Transposon mutagenesis of the Zika virus genome highlights regions essential for RNA replication and restricted for immune evasion. J Virol 91:e00698-17. doi:10.1128/JVI.00698-17. 28515302 PMC5512254

[86] Berto E, Bozac A, Marconi P 2005 Development and application of replication-incompetent HSV-1-based vectors. Gene Ther 12 Suppl 1:S98–S102. doi:10.1038/sj.gt.3302623. 16231061

[87] Wang K, Fredens J, Brunner SF, Kim SH, Chia T, Chin JW 2016 Defining synonymous codon compression schemes by genome recoding. Nature 539:59–64. doi:10.1038/nature20124. 27776354 PMC5321499

[88] Alnaji FG, Holmes JR, Rendon G, Vera JC, Fields CJ, Martin BE, Brooke CB 2019 Sequencing framework for the sensitive detection and precise mapping of defective interfering particle-associated deletions across influenza A and B viruses. J Virol 93:e00354-19. doi:10.1128/JVI.00354-19. 30867305 PMC6532088

[89] Levy DN, Aldrovandi GM, Kutsch O, Shaw GM 2004 Dynamics of HIV-1 recombination in its natural target cells. Proc Natl Acad Sci U S A 101:4204–4209. doi:10.1073/pnas.0306764101. 15010526 PMC384719

[90] Adachi A, Gendelman HE, Koenig S, Folks T, Willey R, Rabson A, Martin MA 1986 Production of acquired immunodeficiency syndrome-associated retrovirus in human and nonhuman cells transfected with an infectious molecular clone. J Virol 59:284–291. doi:10.1128/JVI.59.2.284-291.1986. 3016298 PMC253077

[91] Yanisch-Perron C, Vieira J, Messing J 1985 Improved M13 phage cloning vectors and host strains: nucleotide sequences of the M13mp18 and pUC19 vectors. Gene 33:103–119. doi:10.1016/0378-1119(85)90120-9. 2985470

[92] Vermeire J, Naessens E, Vanderstraeten H, Landi A, Iannucci V, Van Nuffel A, Taghon T, Pizzato M, Verhasselt B 2012 Quantification of reverse transcriptase activity by real-time PCR as a fast and accurate method for titration of HIV, lenti- and retroviral vectors. PLoS One 7:e50859. doi:10.1371/journal.pone.0050859. 23227216 PMC3515444

[93] Livak KJ, Schmittgen TD 2001 Analysis of relative gene expression data using real-time quantitative PCR and the 2^−ΔΔCT^ Method. Methods 25:402–408. doi:10.1006/meth.2001.1262. 11846609

[94] Baer A, Kehn-Hall K 2014 Viral concentration determination through plaque assays: using traditional and novel overlay systems. J Vis Exp 2014:e52065. doi:10.3791/52065. PMC4255882 25407402

[95] Mandell DJ, Lajoie MJ, Mee MT, Takeuchi R, Kuznetsov G, Norville JE, Gregg CJ, Stoddard BL, Church GM 2015 Biocontainment of genetically modified organisms by synthetic protein design. Nature 518:55–60. doi:10.1038/nature14121. 25607366 PMC4422498

