# Supplementary material for: RanDeL-Seq: a High-Throughput Method to Map Viral cis- and trans-Acting Elements
Source: mBio. 2021 Jan 19;12(1):e01724-20. doi: 10.1128/mBio.01724-20 (PMC7845639; doi:10.1128/mBio.01724-20)
Supplement: TABLE S2 [file mBio.01724-20-st002.docx]

| **Name** | **Sequence** |
| --- | --- |
| TN5-F | /5Phos/CTGTCTCTTATACACATCTGCGGCCGC |
| TN5-R | /5Phos/CTGTCTCTTATACACATCTTTAATTAATTCGCTACC |
| BC20v1-F | /5Phos/CCGTCCATGAAGGGTTCGAT |
| BC20v1-R | /5Phos/ACGAATCTGCCGTTGCCATA |
| BC20v1-T | CCGTCCATGAAGGGTTCGATNNNNNNNNNNNNNNNNNNNNTATGGCAACGGCAGATTCGT |
| BC20v2-F | /5Phos/CCGTCCATGAAGCGTTCGAT |
| BC20v2-R | /5Phos/ACGAATCTGCCGTTGCCATA |
| BC20v2-T | CCGTCCATGAAGCGTTCGATNNNNNNNNNNNNNNNNNNNNTATGGCAACGGCAGATTCGT |
| NL43pol-F | GAGACAGGGCAAGAAACAGC |
| NL43pol-R | AACAGGCGGCCTTAACTGTA |
| MS2-F | TCCTGCTCAACTTCCTGTCGAG |
| MS2-R | CAGGTCAAACCTCCTAGGAATG |
| MR766-C-F | TTGGTCATGATACTGCTGATTGC |
| MR766-C-R | CCTCCATGTTCCAAGACAAC |
| A-F | GCGGCCGCTGCAGAATGGGATAGATTGCATCCAGTGCATGCAGGGCCT |
| A-R | GCGGCCGCAGCTGTCCTTTTCTGGCAGCACTATAGGCTGTACTGTCCA |
| B-F | GCGGCCGCTGGACAGTACAGCCTATAGTGCTGCCAGAAAAGGACAGCT |
| B-R | GCGGCCGCGTGAGTTAGCTCACTCATTAGGCACCCCAGGCTTTACACT |
| C-F | AGGCGATTAAGTTGGGTAACGCCAGGGTTTTCCCAGTCACGGCGCGCCGCTCCTCTGGAAAGGTGAAGGGGCAGTAGTAATACAAGAT |
| C-R | GTGAGTTAGCTCACTCATTAGGCACCCCAGGCTTTACACTGGCGCGCCCATTTACCAATACTACTTCTTGTGGGTTGGGGTCTGTGGG |
